# Sampling protocol for the determination of nutrients and contaminants in fish and other seafood – The EAF-Nansen Programme

**DOI:** 10.1016/j.mex.2020.101063

**Published:** 2020-09-12

**Authors:** Amalie Moxness Reksten, Annbjørg Bøkevoll, Sylvia Frantzen, Anne-Katrine Lundebye, Tanja Kögel, Kjersti Kolås, Inger Aakre, Marian Kjellevold

**Affiliations:** Institute of Marine Research, P.O. Box 2029 Nordnes, 5817 Bergen, Norway

**Keywords:** Minerals, Vitamins, Protein, Fatty acids, Metals, Persistent organic pollutants, Food composition data, Food security, Microplastics, Fish

## Abstract

Seafood plays a central role in global food and nutrition security. However, there is a lack of data on the concentration of nutrients and contaminants in fish and other seafood, especially in low- and middle-income countries. In order to assess the potential risks and benefits associated with seafood intake, reliable and up-to-date food composition data is crucial. The quality of food composition data is affected by several factors, such as sampling protocols and the suitability and quality of the methods applied for sample preparation and analysis. In this paper, we describe the sampling methodology and protocols related to the sampling of fish and other seafood and the corresponding analytical methods used to analyse the nutrient and contaminant content of such species. For nutrients, the determination of protein, fat, ash, energy, fatty acids, cholesterol, and amino acids is described, in addition to analyses for determination of the vitamin and mineral content in fish and other seafood. For contaminants, analyses for the determination of organic pollutants and microplastics are described. The methodology described in this paper is used for sampling data through scientific surveys in low- and middle-income countries with research vessel *Dr. Fridtjof Nansen* under the EAF-Nansen Programme. The Programme aims to improve knowledge on the nutritional composition of fish and ensure the fish is safe to consume.

• In this paper, we describe the sampling protocols used for sampling fish and other seafood during scientific surveys under the EAF-Nansen Programme.
• This paper describes the methodology and quality control for analysing nutrients and contaminants in fish and other seafood.

## Graphical abstract

**Figure:**
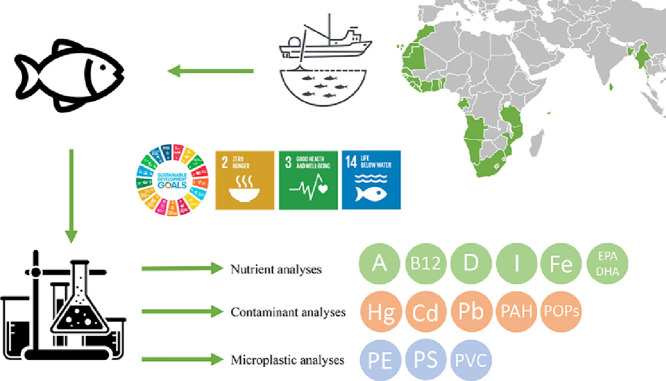

Specifications table

**Table:** 

Subject Area	Environmental Science
More specific subject area	Nutrient and contaminant determination in seafood
Method name	EAF-Nansen Nutrition and Food Safety
Name and reference of original method	NA
Resource availability	NA

Method details

## Background

Fish is an important source of several key nutrients, such as high-quality animal protein, the marine long-chain omega-3 polyunsaturated fatty acids eicosapentaenoic acid (EPA) and docosahexaenoic acid (DHA), vitamin A, vitamin B_12_, vitamin D, iron, zinc, iodine, and selenium [1], [2], [3], [4], [5]. While there is a lack of data on the concentrations of micronutrients in many fish species, existing data indicate that there is considerable variation among species [1,6]. However, fish is also a source of varying levels of contaminants such as metals, persistent organic pollutants (POPs), and plastics accumulated from the marine environment [7,8]. Among contaminants commonly found in fish are mercury (Hg), cadmium (Cd), lead (Pb), arsenic (As), dioxins (polychlorinated dibenzo p-dioxin and dibenzofuran), polychlorinated biphenyls (PCBs), polybrominated diphenyl ethers (PBDE), and per- and polyfluoroalkyl substances (PFAS) [9,10]. Many of these contaminants are classified as either “known” or “probable” human carcinogens, and exposure is associated with several negative health outcomes, including toxic effects on the renal, skeletal, cardiovascular, and neurological systems [11,12]. Furthermore, microplastics are highly persistent in the environment and may accumulate in different marine biota, including seafood, with potential detrimental effects for aquatic life [13]. Currently, toxicity data of sufficient quality are lacking for both micro- and nanoplastics for human risk assessment [14,15].

In order to effectively assess the potential risks and benefits associated with fish intake, it is crucial to have reliable and up-to-date knowledge on the composition of foods, otherwise known as food composition data [16]. Food composition data are quantitative values of the macro- and micronutrients and non-nutrient components in foods, and the quality of the data is affected by several factors, including the sampling (representativity of the samples) and the suitability and quality of the methods applied for sample preparation and analysis [16,17]. In this paper, sampling methods and protocols connected to the sampling of fish and other seafood and the analytical methods used to analyse the nutrient and contaminant content of these, are described in detail. Generally, there is a lack of data on the contents of nutrients and contaminants in fish and seafood, especially in many developing countries, and low-quality data may lead to incorrect research results, erroneous policy decisions (particularly in nutrition, agriculture, and health), misleading food labelling, false health claims, and inadequate food choices [18].

**Fig. 1:**
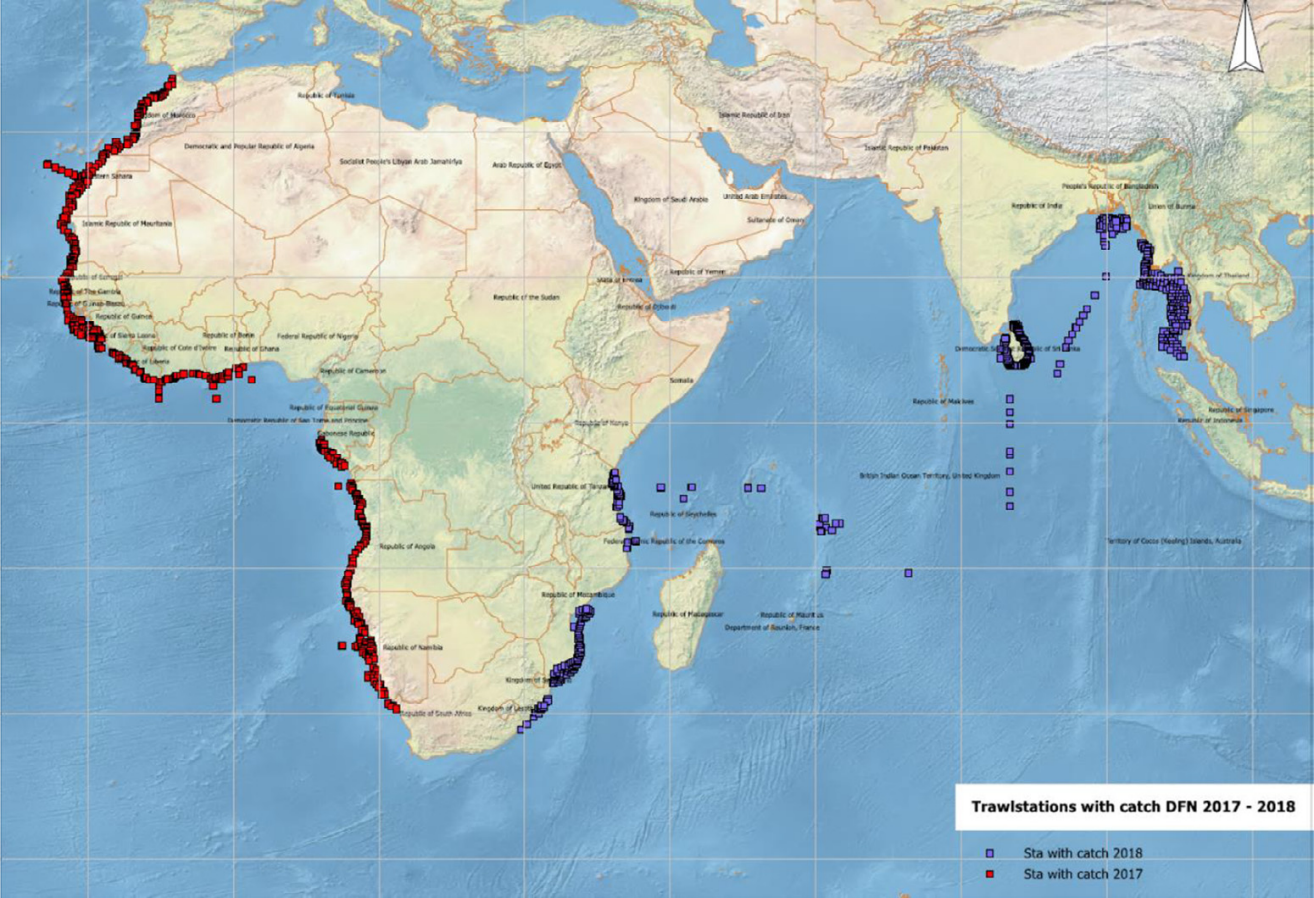
Sampling locations for *Dr. Fridtjof Nansen* (DFN) cruises 2017-2019. Red points illustrate sampling locations during 2017 and 2019 cruises (west coast of Africa and central Africa), whereas blue points illustrate sampling locations in 2018 (east coast of Africa and south of Asia).

The methodology described in this paper was developed for sampling data through scientific surveys with the research vessel (R/V) *Dr. Fridtjof Nansen* as part of the collaboration between the EAF-Nansen Programme and partnering institutions in developing countries. The EAF-Nansen Programme is a partnership between the Food and Agriculture Organization of the United Nations (FAO), the Norwegian Agency for Development Cooperation (Norad), and the Institute of Marine Research (IMR), Norway, for sustainable management of the fisheries of partnering countries. In May 2017, the EAF-Nansen Programme (2017–2021) initiated its first series of cruises incorporating the scientific theme ‘Nutrition and Food Safety’. From 2017-2019, R/V *Dr. Fridtjof Nansen* cruised along the coast of Africa, the Bay of Bengal, and the Indian Ocean (Fig. 1), sampling large numbers of fish and seafood (approximately 6000 samples of 150 different species). The Nutrition and Food Safety theme aim to improve knowledge on the nutritional value and levels of chemical contaminants and biohazards in fish in developing countries [19]. The results may assist national food authorities in evaluating the beneficial health effects of nutrients against potentially negative health effects of contaminants and/or biohazards. If problematic issues are found, measures can be taken to reduce potential risk. Additionally, the results provided from the analyses may facilitate increased export by providing documentation when required by importing countries. The theme has two primary objectives:

1. To improve knowledge on the nutritional composition of fish sampled in selected areas to document the importance of these fish species to food and nutritional security.
2. To ensure fish species sampled in these waters are safe to consume by documenting levels of chemical contaminants and the presence of microplastics.

## Sampling procedures

Sampling of fish was performed during surveys in low- and middle-income countries along the coast of Africa and Asia with R/V *Dr. Fridtjof Nansen*, where pelagic (MultiPelt 624) and demersal trawls (Gisund Super bottom trawl) were continuously towed and placed on deck. For each trawl haul, the fish were sorted according to species, and the species identified by taxonomists on board the vessel. Samples of selected species were collected randomly from the catch shortly after sorting and kept cool in a refrigerator if handling could not begin immediately. Depending on the region, commonly captured and consumed fish species were primarily selected, as advised by the project group and local marine and food scientists on the vessel. For each sampled species, information concerning the time, date, starting and ending position of the trawl haul, the gear type utilised, and the trawling depth(s) were registered. Length (cm) and weight (g) of the fish were measured using a marine measuring board. Fish fork length was measured from the tip of the head to the deepest fork of the caudal fin to the nearest half centimetre, whereas the weight was measured to the nearest gram before further handling/washing. Samples were then separated into two categories for further processing based on their consumption style in local diets: ‘small’ fish species (<25 cm) and ‘large’ fish species (> 25 cm). See protocol 1 in the supplementary material for further details. Additionally, mesopelagic fish species and samples for microplastic analyses were handled separately.

Independently of fish type, whole fish or fillet samples were homogenised on board using a food processor (Braun Multiquick 7 K3000, Kronberg im Taunus, Germany). From the wet homogenate, two subsamples were taken; one for analyses requiring wet sample material and one for analyses where freeze-dried material was possible or preferred. The sub-samples to be kept wet were frozen at -20°C pending shipment to Norway by air cargo. The sub-samples which were to be freeze-dried were frozen at -20°C for at least 12 h before freeze-drying.

## Fish handling: small fish

Fish defined as “small fish” in this project are fish typically consumed whole, with the head, skin, tail, bones, and viscera intact. From each trawl haul (or nearby trawling locations), a total of 150 individual fish were collected. After weight and length measurements, the samples were pooled together to create composite samples consisting of 25 individuals. The fish in three of the composite samples (75 fish) had their head, tail, and viscera removed, whereas the fish in the other three composite samples (75 fish) were kept whole. Each composite sample of 25 fish was then homogenised and two subsamples of the homogenous paste were randomly taken from the grinding container; one sample to be frozen directly and one sample to be freeze-dried, as described below. A detailed description of the protocol for small fish is given in the supplementary material (protocol 2).

## Fish handling: large fish

For large fish, only the fillet is typically consumed and occasionally the liver (or other organs, such as roe, which we did not include). A total of 25 individual fish were sampled from each trawl (or nearby trawling locations). The length, weight, and sex of each individual fish were determined. Using a cutting board, sharp scalpel, and a filleting knife, the fish were eviscerated and filleted and the skin removed, as described in detail in protocol 3 in the supplementary material. The livers of 15 fish were removed and frozen (-20°C) in individual polyethylene containers. The skinless fish fillets were homogenised individually. Five composite samples were prepared by pooling equal amounts of homogenised fillet material from five individual fish (5 × 5). Each composite sample was homogenised again. From both the individual and the composite homogenised fillet samples, two subsamples were frozen at -20°C;one to be kept wet and one to be freeze-dried, respectively.

## Fish handling: mesopelagic fish

Mesopelagic fish are generally small (2–15 cm) and deteriorate quickly and were therefore analysed as composite samples of whole fish. Depending on how many fish or individuals were caught in the trawl, 1–3 composite samples were prepared for each species. The weight of each composite sample was determined, where one composite sample consisted of at least 120 g sample material. The number of fish in each composite sample was counted, allowing a mean weight of each individual fish to be calculated. Each composite sample was then homogenised, and from the homogenate, two subsamples were frozen at -20°C; one to be kept wet and one to be freeze-dried, respectively. A detailed description of the protocol for mesopelagic fish is given in the supplementary material (protocol 4).

## Fish handling: sampling for microplastics analyses

Commonly consumed fish species sampled for microplastic analyses were sampled whole to determine the content of microplastic in whole fish, fish fillet, and fish livers. The larger fish were sampled whole, individually packed in plastic bags, and directly put in the vessel's freezer (-20°C). For smaller fish typically consumed whole, the samples were pooled together to comprise 100 g of sample material and put in pre-cleaned glass jars. Thereafter, the glass jars were frozen upright in the vessel's freezer until shipment to Norway. The methodology is described in further details in protocol 5 in the supplementary material.

## Freeze-drying

The dry matter content of the samples was determined using the freeze-dryer located onboard the ship (Labconco FreeZone 18 l mod. 7750306, Kansas City, MO, USA). After homogenisation, sub-samples of the wet samples were weighed individually on a two-decimal scale and put in separate plastic containers. The samples were frozen (-20°C) for a minimum of 12 h to ensure proper freezing, before they were freeze-dried for 72 h (24 h at -50 °C, immediately followed by 48 h at +25°C, with a vacuum of 0.2–0.01 mbar). After removal from the freeze-dryer, the samples were immediately placed in a desiccator cabinet to avoid drawing humidity from the air. The samples were then weighed once again, enabling the dry matter to be calculated based on the difference in weight of the sample before and after freeze-drying. Calculation of the concentration of dry mass was performed by the following formula:

$$\%\;dry\;matter=\frac{\left(c-d\right)\;x\;100\%}{\left(a-b\right)}$$

Where: a = weight of sample + container before drying (g) b = weight of container before freeze-drying (g) c = weight of the sample + container after drying (g) d = weight of container after freeze-drying (g)

The water/moisture content (%) can then be calculated using this formula:

100%−%drymatter

Freeze-dried samples were then homogenised to fine powder using a knife mill (Retch Grindomix GM 200, Haan, Germany) to enable representative sub-samples to be shipped to Norway for further analyses. Further details of the freeze-drying process and a user guide for the freeze-dryer can be found in protocol 1 in the supplementary material.

## Storage and shipment

Freeze-dried and wet samples were vacuum-sealed and stored in insulated boxes at -20°C in the vessel's freezer until shipment by air cargo to the IMR laboratories in Bergen, Norway. At IMR, the wet samples were stored at -80°C pending analyses, whereas the freeze-dried samples were stored in room temperature in a dark room.

## Analytical quality

All analyses were performed at the IMR laboratories using methods accredited to ISO 17025:2005. Energy and iron analyses are validated methods, but not accredited. The microplastic analyses are not accredited methods either, as the international method development has not developed that far. The first proficiency tests are in progress, but not completed for neither small particle fractions nor seafood matrices. In-house method validation is continuously progressing, and controls are published together with sample analysis. The IMR laboratory regularly participate in national and international proficiency tests to assess the accuracy and precision of the nutrient and contaminant analyses, in addition to the measurement uncertainty of each method. An overview of the validated measurement range and measurement uncertainties (%) of each analytical method are presented in Table 1. Furthermore, Certified Reference Materials (CRM) are analysed at least once a year to check the accuracy and precision of the methods (Table 2), whereas self-produced internal control materials, or reference materials, are included in each sample run for quality control (Table 3).

**Table 1:** Overview of validated measurement range and measurement uncertainty (%) of each analytical method

Analyte	Measurement range^a^	Unit	Measurement uncertainty (%)
Freeze-dried material (moisture)	2–4 4–10 10–99.5	g/100 g	35 20 10
Crude fat	0.1–5 5–15 15–100	g/100 g	12 8 5
Crude protein	0.1–0.7 nitrogen 0.7–16 nitrogen	g/100 g	40 6
Ash	0.1–18	g/100 g	12
Energy		Kcal	2
Fatty acids	0.1–100 % >10 mg/kg	% area percent mg/kg	100 (0.1%)^b^ 50 (0.2–0.5%)^b^ 10 (0.6–2.5%)^b^ 10 (2.5–100 %)^b^
Cholesterol	0.025–50 50–1000 1000–20 000	mg/kg	40 20 15
Amino acids	Hydroxyproline 0.3–5 Histidine 0.7–50 Taurine 0.6–5 Serine 0.5–50 Arginine 0.8–100 Glycine 0.4–50 Aspartic acid 0.7–150 Glutamic acid 0.7–200 Threonine 0.6–50 Alanine 0.4–100 Proline 0.5–100 Lysine 0.7–100 Tyrosine 0.8–50 Methionine 0.7–50 Valine 0.6–100 Isoleucine 0.6–50 Leucine 0.6–100 Phenylalanine 0.8–50	mg/g	10 (20: hydroxyproline, taurine, and tyrosine).
Tryptophan	0.003–0.05 0.05–15	mg/g	20 10
Vitamin A_1_	0.003–100 100–400	mg/kg	20 15
Vitamin A_2_	0.005–100 100–400	mg/kg	20 15
Vitamin E	0.04–1 0.08–1 (α-, β-, and γ-tocotrienol) 1–2000	mg/kg	30 (40 β) 15
Vitamin D_3_ (cholecalciferol)	0.01–0.5 0.5–10 10–40 g/kg	mg/kg	20 15 15
Vitamin B_1_ (thiamine)	0.1–3 3–75	mg/kg	25 15
Vitamin B_2_ (riboflavin)	0.13–75	mg/kg	30
Vitamin B_3_ (niacin)	0.9–10 10–1300	mg/kg	30 20
Vitamin B_6_ (sum pyridoxine, pyridoxal, and pyridoxamine)	0.002–75	mg/kg	15
Vitamin B_9_ (folate)	0.005–8	mg/kg	25
Vitamin B_12_ (cobalamin)	0.001–1.2	mg/kg	30
Calcium (Ca)^a^	35–13000	mg/kg	15
Sodium (Na)^a^	110–6250	mg/kg	15
Potassium (K)^a^	50–17000	mg/kg	15
Magnesium (Mg)^a^	10–3125	mg/kg	15
Phosphorus (P)^a^	3–10000	mg/kg	15
Iron (Fe)^a^	0.1–1 1–1800	mg/kg	40 25 (30: whole fish)
Zinc (Zn)^a^	0.5–5 5–1400	mg/kg	40 20 (30: whole fish)
Selenium (Se)^a^	0.01–0.1 0.1–8	mg/kg	40 25 (30: whole fish)
Cupper (Cu)^a^	0.1–1 1–275	mg/kg	40 25 (30: whole fish)
Arsenic (As)^a^	0.01–0.1 0.1–420	mg/kg	40 25 (30: whole fish)
Lead (Pb)^a^	0.03–0.3 0.3–11	mg/kg	40 25 (30: whole fish)
Cadmium (Cd)^a^	0.005–0.05 0.05–27	mg/kg	40 20 (30: whole fish)
Mercury (Hg)^a^	0.005–0.05 0.05–0.5 0.5–4.6	mg/kg	70 25 20
Iodine (I)^a^	0.04–0.4 0.4-5	mg/kg	40 20
Methylmercury (MeHg) a	3–30 30–200 200–5300	ng/g	34 25 20
PCDD/Fs	0.008–8000^c^	pg/g	20–40
Non-ortho PCBs	0.03–3200^c^	pg/g	25–45
Mono-ortho PCBs	2–25600^c^	pg/g	30–50
PCB6	0.01–320^c^	ng/g	30–40
PBDEs	0.001–500^c^	ng/g	30–50
PAH Benz(a)anthracene Benzo(a)pyrene Benzo(b)fluoranthene Benzo(c)fluorene Benzo(g.h.i)perylene Benzo(j)fluoranthene Benzo(k)fluoranthene Chrysene Cyclopenta(c.d)pyrene Dibenz(a.h)anthracene Indeno(1.2.3.-cd)pyrene 5-methylchrysene	0.15	ng/g	30
Dibenzo(a.e)pyrene Dibenzo(a.h)pyrene Dibenzo(a.i)pyrene Dibenzo(a.l)pyrene	0.75	ng/g	60

a Range by dry weight. The lowest value represents the limit of quantification (LOQ).

b The measurement uncertainty for fatty acids is divided into four levels, depending on the area percentage of the fatty acid. The area percentage is presented within the parentheses, and the corresponding measurement uncertainty is presented in front of the parentheses.

c Weight dependent. Abbreviations: As: arsenic, Ca: calcium, Cd: cadmium, Cu: cupper, Fe: iron, Hg: mercury, K: potassium, MeHg: methylmercury, Mg: magnesium, Na: sodium, P: phosphorus, PAH: polycyclic aromatic hydrocarbons, Pb: lead, PBDE: polybrominated diphenyl ethers, PCB: polychlorinated biphenyls, PCDD: polychlorinated dibenzodioxins, PCDF: polychlorinated dibenzofurans, Se: selenium, Zn: zinc.

**Table 2:** Overview of Certified Reference Materials (CRM)

Analyte	Reference material(s) a^,^^b^^,^^c^^,^^d^^,^^e^	Certified value ± U = 2*u	Analysed value ± U = 2*u	Unit	Mean accuracy (%)
Freeze-dried material (moisture)	ERM-BD017a	75.8 ± 2.0	75.8 ± 2.0	g/100 g	101
Crude fat	SMRD 2000	14.3 ± 0.5	13.8 ± 0.26	g/100 g	97
Crude protein	ERM-BD017a	0.746 ± 0.04	0.72 ± 0.03	g/100 g	97
Ash	RM LGC 7107 SMRD 2000	2.65 ± 0.1 1.76 ± 0.07	2.67 ± 0.05 1.8	g/100 g	101 102
Energy	Benzoic acid tablet	26454	26474 ± 160	j/g	100
Fatty acids	SRM2387 SRM1544	0.024–23.38 0.1–11.64	0.03–25 0.1–13	g/100 g g/kg	76–121 80–135
Cholesterol	SRM 1544 SRM1845	148.3 ± 9.4 18640 ± 390	147 ± 19 17564 ± 1611	mg/kg	100 96
Histidine Taurine Serine Arginine Glycine Aspartic acid Glutamic acid Threonine Alanine Proline Lysine Tyrosine Methionine Valine Isoleucine Leucine Phenylalanine	SRM1849a	3.15 ± 0.6 0.366 ± 0.02 7.2 ± 0.3 4 ± 0.29 2.4 ± 0.19 10.7 ± 0.57 25.9 ± 2.7 6.4 ± 0.22 4.55 ± 0.21 11.95 ± 0.86 10.1 ± 0.71 5.1 ± 0.43 4.57 ± 0.7 7.6 ± 1.1 6.6 ± 0.71 12.61 ± 0.5 5.8 ± 0.21	2.98 ± 0.21 0.33 ± 0.03 7.0 ± 0.4 3.9 ± 0.25 2.5 ± 0.17 11.3 ± 0.55 26.3 ± 1.5 6.4 ± 0.21 4.59 ± 0.16 11.85 ± 0.28 10.9 ± 0 75 5.1 ± 0.51 4.54 ± 0.3 8.1 ± 0.6 6.9 ± 0.33 12.73 ± 0.31 5.9 ± 0.44	mg/kg	95 91 97 98 103 106 101 100 101 99 108 101 99 107 104 101 101
Tryptophan	CRM 2387 CRM 1849a	0.21 ± 0.06 0.184 ± 0.01	0.22 ± 0.05 0.177 ± 0.01	g/100 g	103 96
Vitamin A_1_	SRM2383 SRM1849a	0.80 ± 0.15 7.68 ± 0.23	0.71 ± 0.09 7.57 ± 0.68	mg/kg	89 99
Vitamin A_2_	N/A^f^	N/A^f^	N/A^f^	N/A	N/A^f^
Vitamin E	SRM1849a α-tocopherol SRM2387 α-tocopherol SPM 2387 β +γ-tocopherol SRM 2387 δ-tocopherol SRM1950 α-tocopherol SRM1950 β +γ-tocopherol	219 ± 16 108 ± 11 100 ± 19 10 ± 3 8.01 ± 0.22 1.67 ± 0.16	197 ± 15 90 ± 10 92 ± 11 7.6 ± 1 7.9 ± 0.2 1.62 ± 0.01	mg/kg	90 84 92 76 98 97
Vitamin D_3_ (cholecalciferol)	CRM421 SRM1849a	0.143 ± 0.008 0.111 ± 0.02	0.146 ± 0.029 0.12 ± 0.03	mg/kg	102 108
Vitamin B_1_ (thiamine)	CRM383 SRM1849a	2.2 12.57 ± 0.98	1.92 ± 0.32 13.9 ± 2.86	mg/kg	87 111
Vitamin B_2_ (riboflavin)	SRM1546B SRM1849a	2.0 ± 0.59 20.37 ± 0.52	1.7 ± 0.4 19.96 ± 3.5	mg/kg	82 98
Vitamin B_3_ (niacin)	CRM 383B SRM1849a	17 109 ± 10	15.4 ± 2.9 102 ± 18	mg/kg	91 94
Vitamin B_6_ (sum pyridoxine, pyridoxal, and pyridoxamine)	SRM1849a CRM2387	13.46 ± 0.93 4.66 ± 0.62	13.97 ± 0.87 4.69 ± 0.64	mg/kg	104 101
Vitamin B_9_ (folate)	SRM1849a	2.29 ± 0.06	2.41 ± 0.41	mg/kg	105
Vitamin B_12_ (cobalamin)	SRM1849a SRM1546a	0.0482 ± 0.0085 0.0055 ± 0.002	0.048 ± 0.016 0.0053 ± 0.001	mg/kg	100 101
Calcium (Ca)	SRM 1577c SRM BD-150	131 ± 10 13900 ± 800	132 ± 15 13076 ± 1470	mg/kg	101 94
Sodium (Na)	SRM 1577c SRM BD-150	2033 ± 64 4180 ± 190	1959 ± 153 3867 ± 351	mg/kg	96 93
Potassium (K)	SRM 1577c SRM BD-150	10230 ± 640 17000 ± 700	10258 ± 789 16804 ± 1609	mg/kg	100 99
Magnesium (Mg)	SRM 1577c SRM BD-150	620 ± 42 1260 ± 100	604 ± 61 1163 ± 117	mg/kg	97 92
Phosphorus (P)	SRM 1577c SRM BD-150	11750 ± 270 11000 ± 600	10918 ± 951 10186 ± 1041	mg/kg	93 93
Iron (Fe)	NIST1566b NRC Tort 3	205.8 ± 6.8 179 ± 8	187.4 ± 15.9 159 ± 13	mg/kg	90 89
Zinc (Zn)	NIST1566b NRC Tort 3	1424 ± 46 136 ± 6	1382 ± 145 128 ± 9	mg/kg	97 94
Selenium (Se)	NIST1566b NRC Tort 3	2.06 ± 0.15 10.9 ± 1.0	1.97 ± 0.17 10.1 ± 0.73	mg/kg	96 93
Cupper (Cu)	NIST1566b NRC Tort 3	71.6 ± 1.6 497 ± 22	64.5 ± 7.2 428 ± 54	mg/kg	90 86
Arsenic (As)	NIST1566b NRC Tort 3	7.65 ± 0.65 59 5 ± 3.8	7.39 ± 0.22 65.1 ± 4.5	mg/kg	97 109
Lead (Pb)	NIST1566b NRC Tort 3	0.31 ± 0.01 0.23 ± 0.02	0.30 ± 0.01 0.20 ± 0.02	mg/kg	98 91
Cadmium (Cd)	NIST1566b NRC Tort 3	2.48 ± 0.08 42.3 ± 1.8	2.49 ± 0.08 40.4 ± 3.2	mg/kg	100 95
Mercury (Hg)	NIST1566b NRC Tort 3	0.04 ± 0.0013 0.29 ± 0.02	0.03 ± 0,00 0.26 ± 0.03	mg/kg	85 89
Iodine (I)	ERM-BD-150 ERM-BB-422	1.73 ± 0.14 1.4 ± 0.4	1.52 ± 0.15 1.26 ± 0.20	mg/kg	88 89
Methyl mercury (MeHg)	SRM1566b NRC Tort 3 BCR-627	13.2 ± 0.35 137 ± 12 5117 ± 158	17.4 ± 5.5 127 ± 19 5142 ± 307	ng/g	124 93 100
PCDD/Fs 2378-TCDD 12378-PeCDD 123478-HxCDD 123678-HxCDD 123789-HxCDD 1234678-HpCDD OCDD 2378-TCDF 12378-PeCDF 23478-PeCDF 123478-HxCDF 123678-HxCDF 123789-HxCDF 234678-HxCDF 1234678-HpCDF 1234789-HpCDF OCDF	FHI 2015 Interlab. test C	0.39 ± 0.184 0.68 ± 0.3 0.037 ± 0.022 0.27 ± 0.13 0.02 ± 0.013 0.036 ± 0.026 0.12 ± 0.094 5.7 ± 2.4 0.86 ± 0.46 4.5 ± 1.92 0.12 ± 0.068 0.17 ± 0.094 0.0056 ± 0.007 0.15 ± 0.076 0.016 ± 0.0166 0.005 ± 0.0078 0.021 ± 0.026	0.4 ± 0.1 0.6 ± 0.0 0.03 ± 0.009 0.25 ± 0.1 0.01 ± 0.004 0.02 ± 0.022 0.1 ± 0.06 5.08 ± 0.4 0.76 ± 0.17 4.08 ± 0.7 0.1 ± 0.002 0.14 ± 0.04 N/A 0.15 ± 0.025 0.01 ± 0.001 N/A N/A	pg/g	91 106 77 94 67 69 87 89 89 91 84 84 N/A 102 45 N/A N/A
Non-ortho PCBs PCB-77 PCB-81 PCB-126 PCB-169	FHI 2015 Interlab. test C	106 ± 60 2.3 ± 1.6 48 ± 20 12 ± 5.2	95.3 ± 11.7 2.32 ± 0.4 41.7 ± 5.3 10.4 ± 1.1	pg/g	90 101 87 87
Mono-ortho PCBs PCB-105 PCB-114 PCB-118 PCB-123 PCB-156 PCB-157 PCB-167 PCB-189	FHI 2015 Interlab. test C	1972 ± 982 120 ± 66 5627 ± 2936 64 ± 58 876 ± 344 207 ± 74 469 ± 226 93 ± 34	1663 ± 127 104 ± 24 4953 ± 659 83.4 ± 51 754 ± 99 182 ± 21 398 ± 43.3 79.8 ± 13.8	pg/g	84 87 88 130 86 88 85 86
ndl-PCBs PCB-28 PCB-52 PCB-101 PCB-138 PCB-153 PCB-180 *PCB-31*	FHI 2015 Interlab. test C	703 ± 568 1593 ± 968 6220 ± 3664 10606 ± 7656 15316 ± 9744 4711 ± 2678 (*not part of test*	834 ± 597 1498 ± 267 6072 ± 2157 11266 ± 6224 17128 ± 6733 4578 ± 2045	pg/g	119 94 98 106 112 97
PBDEs PBDE 28 PBDE 47 PBDE 99 PBDE 100 PBDE 153 PBDE 154 PBDE 183 *PBDE 35* *PBDE 49* *PBDE 66* *PBDE 71* *PBDE 75* *PBDE 77* *PBDE 85* *PBDE 118* *PBDE 119* *PBDE 138*	FHI 2015 Interlab. test C	62 ± 26 1572 ± 662 333 ± 96 416 ± 130 93 ± 22 262 ± 110 2.9 ± 2.4 *Not part of test*	56.11 ± 21.4 1450 ± 805 326 ± 130 340 ± 113 73 ± 8 206 ± 30 N/A	pg/g	90 96 98 93 81 82
PAH Benz(a)anthracene Benzo(a)pyrene Benzo(b)fluoranthene Benzo(g,h,i)perylene Benzo(j)fluoranthene Benzo(k)fluoranthene Chrysene Indeno(1,2,3,-cd)pyrene	SRM 2974a	31.1 ± 3.9 9.73 ± 0.4 41.5 ± 2.6 23.7 ± 2.2 21.4 ± 1.1 18.95 ± 0.5 85.1 ± 1.1 14.9 ± 4.5	26.3 ± 2.9 6.6 ± 0.2 42.9 ± 3.3 20.9 ± 2.0 19.4 ± 2.4 18.0 ± 0.8 89.0 ± 7.31 14.0 ± 0.9	ng/g	85 68 103 88 91 95 105 94

a ERM-BD017a (sponge cake), SRMD2000 (meat) and LGC7107 (Madeira cake) LGC, Teddington Middlesex, UK.

b CRM 1556b (oyster tissue), SRM2387 (peanut butter), SRM1544 (diet composite), SRM1845 (whole egg powder), SRM1849 (infant/adult nutritional formula), SRM2383 (baby food composite), SRM1950 (frozen human plasma), and CRM1556b (oyster tissue), National Institute of Standards and Technology, Gaithersburg, MD, USA.

c TORT-3 (lobster hepatopancreas), National Research Council, Ontario, Canada.

d CRM383B (haricots verts), ERM-BD150 (skimmed milk powder), and ERM-BB422 (fish muscle), and BCR-627 (tuna fish), Joint Research Centre, Geel, Belgium.

e Benzoic acid tablet (benzoic acid), Parr Instrument Company, Moline, IL, USA.

f No certified reference materials (CRM) available for vitamin A_2_. Abbreviations: As: arsenic, Ca: calcium, Cd: cadmium, Cu: cupper, Fe: iron, Hg: mercury, K: potassium, MeHg: methylmercury, Mg: magnesium, N/A: not available, Na: sodium, P: phosphorus, PAH: polycyclic aromatic hydrocarbons, Pb: lead, PBDE: polybrominated diphenyl ethers, PCB: polychlorinated biphenyls, PCDD: polychlorinated dibenzodioxins, PCDF: polychlorinated dibenzofurans, Se: selenium, Zn: zinc.

**Table 3:** Overview of internal control materials

Analyte	Control material	Analysed value ± U = 2*u	2RSD (%)	Unit
Freeze-dried material (moisture)	Salmon muscle	37.3 ± 0.32	1	g/100 g
Crude fat	Fish feed	25.3 ± 1.26	5	g/100 g
Crude protein	TET003RM canned meat	8.38 ± 0.4 (nitrogen)	5	g/100 g
Ash	Fish feed	12.06 ± 0.36	3	g/100 g
Energy	Benzoic acid tablet	26474 ± 160	1	J/g
Fatty acids	Salmon liver	Area % 16:0: 8.8 ± 0.2 18:1n-9: 35.8 ± 0.6 20:5n-3: 4.7 ± 0.2 mg/g 16:0: 4.9 ± 0.2 18:1n-9: 20.0 ± 1.2 20,5n-3: 2.6 ± 0.2 Total fatty acids mg/g: 55.2 ± 2.8	2 2 2 5 6 5 5	g/100 g g/kg
Cholesterol	SRM 1544 diet composite SRM1845 whole egg powder	147 ± 19 17564 ± 1611	18 9	mg/kg
Hydroxyproline Histidine Taurine Serine Arginine Glycine Aspartic acid Glutamic acid Threonine Alanine Proline Lysine Tyrosine Methionine Valine Isoleucine Leucine Phenylalanine	Granulate from cod	104 ± 019 18.5 ± 1.8 1.98 ± 0.21 42.9 ± 3.0 60.4 ± 5.7 37.8 ± 3.1 105.5 ± 8.6 159.2 ± 12.0 4.0 ± 3.0 54.8 ± 3.9 31.3 ± 1.6 93.9 ± 8.4 34.9 ± 4.4 31.8 ± 2.6 47.3 ± 2.6 45.0 ± 3.3 79.4 ± 5.3 38.1 ± 3.8	18 10 11 7 9 8 8 8 7 7 5 9 13 8 5 7 7 10	mg/g
Tryptophan	SMRD2000 Meat Casein	3.25 ± 0.16 11,.1 ± 0.36	5 3	mg/g
Vitamin A_1_	Mixed salmon muscle and liver	3.1 ± 0.40 (all-trans retinol) 6.0 ± 0.56 (A_1_)	13 9	mg/kg
Vitamin A_2_	Mixed salmon muscle and liver	5.7 ± 0.96	17	mg/kg
Vitamin E	Salmon muscle	37.66 ± 5 (α-tocopherol) 0.10 ± 0.04 (β-tocopherol) 15.7 ± 2 (γ-tocopherol) 0.26 ± 0.06 (δ-tocopherol) 0.47 ± 0.4 alfa	13 36 13 23 24	mg/kg
Vitamin D_3_ (cholecalciferol)	Enriched salmon muscle	0.31 ± 0.04	14	mg/kg
Vitamin B_1_ (thiamine)	Salmon muscle	2.09 ± 0.32	15	mg/kg
Vitamin B_2_ (riboflavin)	Salmon muscle	1.01 ± 0.2	20	mg/kg
Vitamin B_3_ (niacin)	Fish meal	138.3 ± 20.8	15	mg/kg
Vitamin B_6_ (sum pyridoxine, pyridoxal, and pyridoxamine)	Salmon muscle	6.45 ± 0.88	14	mg/kg
Vitamin B_9_ (folate)	Fishmeal	0.48 ± 0.10	20	mg/kg
Vitamin B_12_ (cobalamin)	Fishmeal	0.32 ± 0.08	25	mg/kg
Calcium (Ca)	SRM 1577c Bovine liver SRM BD-150 Milk powder	132 ± 15 13076 ± 1470	12 12	mg/kg
Sodium (Na)	SRM 1577c Bovine liver SRM BD-150 Milk powder	1959 ± 153 3867 ± 351	8 9	mg/kg
Potassium (K)	SRM 1577c Bovine liver SRM BD-150 Milk powder	10258 ± 789 16804 ± 1609	8 10	mg/kg
Magnesium (Mg)	SRM 1577c Bovine liver SRM BD-150 Milk powder	604 ± 61 1163 ± 117	10 10	mg/kg
Phosphorus (P)	SRM 1577c Bovine liver SRM BD-150 Milk powder	10918 ± 951 10186 ± 1041	9 10	mg/kg
Iron (Fe)	NIST1566b Oyster tissue NRC Tort 3 Lobster hepatopancreas	187.4 ± 15.9 159 ± 13	9 8	mg/kg
Zinc (Zn)	NIST1566b Oyster tissue NRC Tort 3 Lobster hepatopancreas	1382 ± 145 128 ± 9	11 7	mg/kg
Selenium (Se)	NIST1566b Oyster tissue NRC Tort 3 Lobster hepatopancreas	1.97 ± 0.17 10.1 ± 0.73	8 7	mg/kg
Cupper (Cu)	NIST1566b Oyster tissue NRC Tort 3 Lobster hepatopancreas	64.5 ± 7.2 428 ± 54	11 13	mg/kg
Arsenic (As)	NIST1566b Oyster tissue NRC Tort 3 Lobster hepatopancreas	7.39 ± 0.22 65.1 ± 4.5	6 7	mg/kg
Lead (Pb)	NIST1566b Oyster tissue NRC Tort 3 Lobster hepatopancreas	0.30 ± 0.01 0.20 ± 0.02	8 8	mg/kg
Cadmium (Cd)	NIST1566b Oyster tissue NRC Tort 3 Lobster hepatopancreas	2.49 ± 0.08 40.4 ± 3.2	6 8	mg/kg
Mercury (Hg)	NIST1566b Oyster tissue NRC Tort 3 Lobster hepatopancreas	0.03 ± 0.007 0.26 ± 0.03	22 12	mg/kg
Iodine (I)	ERM-BD150 Milk powder ERM-BB422 Fish muscle	1.52 ± 0.15 1.26 ± 0.20	10 16	mg/kg mg/kg
Methyl mercury (MeHg)	SRM1566b Oyster tissue NRC Tort 3 Lobster BCR-627 Tuna fish	17.4 ± 5.5 127 ± 19 5142 ± 307	32 15 6	mg/g
PCDD/Fs 2378-TCDD 12378-PeCDD 123478-HxCDD 123678-HxCDD 123789-HxCDD 1234678-HpCDD OCDD 2378-TCDF 12378-PeCDF 23478-PeCDF 123478-HxCDF 123678-HxCDF 123789-HxCDF 234678-HxCDF 1234678-HpCDF 1234789-HpCDF OCDF	Freeze-dried salmon (2015-1388), spiked	4 ± 0.75 17.38 ± 3.39 18 ± 2.5 17.8 ± 2.83 15.47 ± 4.37 17.27 ± 2.45 34.58 ± 3.74 4.74 ± 1.08 16.88 ± 1.99 17.57 ± 2.19 18.32 ± 2.67 17.93 ± 3.46 17.46 ± 4.34 18.59 ± 3.53 18.39 ± 2.96 18.14 ± 2.95 34.14 ± 7.46	18 20 14 16 28 14 10 22 12 12 14 10 24 18 16 16 22	TEQ pg/g
Non-ortho PCBs PCB-77 PCB-81 PCB-126 PCB-169	Freeze-dried salmon (2015-1388)	21.02 ± 2.25 0.98 ± 0.17 8.05 ± 1.61 3.06 ± 1.77	10 16 20 50	TEQ pg/g
Mono-ortho PCBs PCB-105 PCB-114 PCB-118 PCB-123 PCB-156 PCB-157 PCB-167 PCB-189	Freeze-dried salmon (2015-1388)	432 ± 51 28 ± 6 1455 ± 185 19 ± 12 150 ± 24 43 ± 10 100 ± 23 23 ± 8	12 22 12 64 16 24 24 36	pg/g
ndl-PCBs PCB-28 PCB-52 PCB-101 PCB-138 PCB-153 PCB-180 PCB-31	Freeze-dried salmon (2015–1388)	337 ± 27 977 ± 134 1944 ± 228 2558 ± 424 4497 ± 446 1324 ± 115 294 ± 24	8 14 12 16 10 8 16	pg/g
PBDEs PBDE 28 PBDE 47 PBDE 99 PBDE 100 PBDE 153 PBDE 154 PBDE 183	Freeze-dried salmon (2015-1388), spiked	1.17 ± 0.07 2.05 ± 0.2 1.31 ± 0.06 1.37 ± 0.09 1.23 ± 0.15 1.29 ± 0.08 1.14 ± 0.15	6 10 4 6 12 6 14	ng/g
PBDE 35 PBDE 49 PBDE 66 PBDE 71 PBDE 75 PBDE 77 PBDE 85 PBDE 118 PBDE 119 PBDE 138		0.73 ± 0.11 1.32 ± 0.21 1.14 ± 0.15 1.02 ± 0.08 1.21 ± 0.11 1.1 ± 0.1 0.99 ± 0.12 1.12 ± 0.08 1.08 ± 0.11 1.13 ± 0.08	14 16 14 8 8 8 12 6 10 6	
PAH Benz(a)anthracene Benzo(a)pyrene Benzo(b)fluoranthene Benzo(c)fluorene Benzo(g,h,i)perylene Benzo(j)fluoranthene Benzo(k)fluoranthene Chrysene Cyclopenta(c,d)pyrene Dibenz(a,h)anthracene Indeno(1,2,3,-cd)pyrene 5-methylchrysene Dibenzo(a,e)pyrene Dibenzo(a,h)pyrene Dibenzo(a,i)pyrene Dibenzo(a,l)pyrene	Salmon muscle	4.02 ± 0.13 3.86 ± 0.46 3.89 ± 0.26 3.90 ± 0.84 4.01 ± 0.43 4.13 ± 0.39 3.84 ± 0.32 4.10 ± 0.41 3.91 ± 0.59 3.92 ± 0.31 4.03 ± 0.46 3.79 ± 1.01 3.69 ± 0.95 1.59 ± 0.83 3.23 ± 1.19 3.50 ± 0,84	6 12 7 22 11 9 8 10 15 8 11 6 26 52 37 24	ng/g

Abbreviations: As: arsenic, Ca: calcium, Cd: cadmium, Cu: cupper, Fe: iron, Hg: mercury, K: potassium, MeHg: methylmercury, Mg: magnesium, Na: sodium, P: phosphorus, PAH: polycyclic aromatic hydrocarbons, Pb: lead, PBDE: polybrominated diphenyl ethers, PCB: polychlorinated biphenyls, PCDD: polychlorinated dibenzodioxins, PCDF: polychlorinated dibenzofurans, Se: selenium, TEQ: toxic equivalency factor, Zn: zinc.

## Determination of crude fat and protein, ash, energy, cholesterol, fatty acids, and amino acids

For the determination of fat (crude fat), 1–5 g (depending on expected amount of fat) sample material was weighed into a 50 ml screw cap bottle, before 30 ml of ethyl acetate/isopropyl alcohol (70/30) was added to extract the fat. The bottle was corked and shaken for 2 h in a shaker, before the sample was filtered through a folding filter in a 100 ml Erlenmeyer flask. Between 5 and 10 ml (depending on the amount of fat in the sample) was then pipetted into a tarred evaporating bowl, and the bowl was placed in an oven at 70°C until the water had evaporated (approximately 16 h). The bowl was cooled to room temperature in a desiccator and the amount of fat weighed. The method is based on a Norwegian Standard [20].

Crude protein was calculated from total nitrogen which was determined by burning the material in pure oxygen gas in a combustion tube (Leco FP 628, Leco Corporation, Saint Joseph, MI, USA) at 950°C. Nitrogen was detected with a thermal conductivity detector (TCD, Leco Corporation, Saint Joseph, MI, USA) and the content of nitrogen was calculated from an estimated average of 16% nitrogen per 100 g protein. The following formula was used: g nitrogen/100 g x 6.25 = g protein/100 g, in accordance with the method accredited by the Association of Official Agricultural Chemists (AOAC) [21].

Ash is defined as the inorganic residue obtained after removal of moisture and organic matter by heat treatment. Analysis of ash content was performed according to the Nordic Committee on Food Analysis (NMKL) Method 23.3 [22]. Depending on the matrix, 1–5 g of homogenised sample material was weighed into a pre-weighed quartz crucible. The sample was placed in a cold muffle oven (Thermolyne F 30430 CM, Thermo Fisher Scientific, Waltham, MA, USA) where the temperature was gradually increased to 550 ± 5°C and ashed overnight (approximately 16–18 h) at normal pressure. The sample was cooled in a desiccator before being weighed again to determine the weight loss.

Energy was measured using an Automatic Isoperibol Calorimeter (Parr Calorimeter 6400, Moline, IL, USA). One gram of freeze-dried sample material was pressed to a tablet in a pellet press and thereafter burned in a high-pressure oxygen atmosphere within a metal pressure vessel. The energy released by the combustion was absorbed within the calorimeter and the resulting temperature change was recorded. The gross energy was calculated from the calorimeter's heat capacity and the temperature increase in the water, as temperature increase is a direct expression of the sample's calorific value and of the amount of energy present in the sample. The temperature change was converted into the total energy content of the fish, expressed in kilojoules (kJ). Sample preparation, the use of the instrument, and the calculations performed are described in detail in the operating instructions manual for the instrument [23].

The sample preparation for cholesterol was based on the method for analysis of cholesterol in milk products described by Fletouris et al. (1998), [24], but by using sodium hydroxide (NaOH)/methanol instead of potassium hydroxide (KOH)/methanol. Prior to weighing the sample, 1 ml of internal standard α-5 cholestane (0.2 mg/ml, Sigma Aldrich, purity ≥ 99%, Art. No. C8667) was added to the sample preparation tube [25]. Cholesterol was analysed on a Thermo Trace 2000 GC (Thermo Fisher Scientific, Waltham, MA, USA), with instrument conditions as described by Araujo et al (2006); [25]. The software Chromeleon® version 7.2 (Thermo Fisher Scientific, Waltham, MA, USA) was used for integration and calculation of the cholesterol content.

For analysis of fatty acids, lipids from the samples were extracted according to Folch et al. (1957); [26]. After filtering, the remaining samples were saponified with 0.5 M NaOH and methylated using 12% Boron trifluoride (BF3) in methanol at 100°C. After cooling the solution, the methyl ester was extracted with hexane. The fatty acid composition of total lipids was analysed as previously described by Lie & Lambertsen (1991); [27], and Torstensen et al. (2004); [28]. Methyl esters were separated using a Perkin Elmer Auto System XL2000 gas chromatograph, (‘cold on column’ injection; 60°C for 1 min, 25°C min−1, 160°C for 25 min, 25°C min−1, 190°C for 17 min, 25°C min−1, 220°C for 6 min), equipped with a 50 m CP-sil 88 (Chromopack Ltd., Middelburg, The Netherlands) fused silica capillary column (id: 0.32 mm). The methyl esters were detected on a Flame Ionization Detector (FID, Perkin Elmer, Waltham, MA, USA) and peaks were identified by retention time using standard mixtures of methyl esters (Nu-Chek, Elysian, USA), thus determining the fatty acid composition (area %). All samples were integrated using Chromeleon^Ⓡ^ connected to the gas liquid chromatograph (GLC). The amount of fatty acids per gram sample was calculated using 19:0 methyl ester as an internal standard.

For the determination of amino acids, the sample was added to an internal standard (Norvaline, Sigma-Aldrich, purity 99%, Art. No. N7502) and hydrolysed in hydrochloric acid. After hydrolysis, the hydrochloric acid was removed from the samples using a vacuum centrifuge. The samples were added water and filtered before derivatisation with AccQ- Tag reagent [29]. The derivative was analysed using Ultra Performance Lipid Chromatography (UPLC, reverse phase) and ultraviolet (UV) detection at 260 nm (Waters Acquity UPLC System, Waters, Milford, MA, USA). The samples were integrated using the software Empower version 3 (Waters, Milford, MA, USA). Quantification was determined using internal and external standard curves. Sample preparation, use of the instrument, and calculations performed are described in Waters, AccQ-Tag^TM^ Method 715001320, REV D [29].

For the determination of the amino acid tryptophan, the protein in the sample (1.2 g) was hydrolysed in a pressure cooker for 20 h at 110°C with barium hydroxide (8.4 g/15 ml Milli-Q water). The hydrolysate was cooled to room temperature and 4 ml of hydrochloric acid solution (1000 ml HCL/200 ml Milli-Q water) was added. The pH was adjusted to 3–4 with the same hydrochloric acid solution and 2 M NaOH. The solution was diluted in a 50 ml volumetric flask with water. Prior to determination by analytical high-performance liquid chromatography (HPLC, reverse phase) using an UV detector 280 nm (Agilent 1290 Infinity system, Agilent Technologies, PDA, Santa Clara, CA, USA), the solution was first filtered through a folding filter and 2 ml of the solution was filtered with a 0.45 µm Millipore syringe filter (instrument conditions: column, Poroshell 120 EC-C18, 3 × 50 mm, 2.7 µm.; mobile phase: 0.0085 M NaOAc; flow rate: 0.5 ml/min). The samples were integrated using the software Empower, and the tryptophan content was calculated by external calibration (standard curve). The principle of the method is based on a previously described method [30,31].

## Determination of vitamins

For the determination of the vitamin A_1_ (sum of all trans-retinol and 13-, 11-, 9 cis retinol) and vitamin A_2_ (3,4 didehydro-all-trans-retinol) content, weighed sample material (0.2–1 g depending on the matrix) were mixed with 4 ml ethanol, one spatula tip of pyrogallol, one spatula tip of ascorbic acid, 0.5 ml saturated ethylenediaminetetraacetic acid (EDTA), and 0.5 ml 20% KOH. The solution was saponified in a block-heater (100°C) for 20 min. After saponification, the mixture was cooled, 1 ml distilled water and 3 ml n-hexane were added, the solution was mixed, centrifuged, and the free vitamin A were extracted in the hexane face. The hexane phase (upper layer) was transferred with a Pasteur-pipette into a 10 ml sample tube (vitamin A was totally extracted with 3 × 3 ml of n-hexane). The hexane extract was evaporated to dryness at ambient temperature under a N_2_ atmosphere and added appropriate amounts of n-hexane (depending on the matrix) for analysis by HPLC. Vitamin A_1_ (sum of all trans-retinol and 13-, 11-, 9 cis retinol) and vitamin A_2_ (3,4 didehydro-all-trans-retinol) were separated and determined by HPLC (normal phase) using a Photo Diode Array detector EM 326 nm (HPLC 1260 system Agilent Technologies, PDA, Santa Clara, CA, USA; instrument conditions: column, Kromasil 100–3.5 sil, 150 × 2.1 mm; mobile phase: 11% tert-Butyl methyl ether (tbme)/89% n-Heptane; flow rate: 0.5 ml/min). All samples were integrated using Chromeleon^Ⓡ^ where the content of all-trans-retinol was calculated by external calibration (standard curve) and the content of the other vitamin A forms were calculated based on the external calibration curve for all-trans-retinol multiplied by a correction factor (NS-EN 12823-1). The method is based on a method previously described by the Comitè Europèen de Normalisation (CEN) [32].

Vitamin B_1_ (thiamine) was released from the sample by acid extraction, hydrolysis, and enzyme treatment and further post-column derivation (reverse phase) of thiamine to thiochromone, prior to detection by a fluorescence detector (Ex366 nm, Em435 nm; Agilent 1100 HPLC system, Agilent Technologies, PDA, Santa Clara, CA, USA). All samples were integrated using Chromeleon^Ⓡ^, and the vitamin B_1_ content was calculated by external calibration (standard curve; [33]). Vitamin B_2_ (riboflavin) was released from the sample by acid extraction, hydrolysis, and enzyme treatment and determined by HPLC (reverse phase) using a fluorescence detector (Ex468 nm, Em5204 nm; Agilent 1100 HPLC system, Agilent Technologies, PDA, Santa Clara, CA, USA). All samples were integrated using Chromeleon^Ⓡ^, and the vitamin B_2_ content was calculated by external calibration (standard curve; [34]). Vitamin B_3_ (niacin) was released from the sample by extraction (autoclaving in sulfuric acid) and mixed with growth medium, added to the microorganism *Lactobacillus plantarum* (ATCC 8014), and incubated at 37°C for 22 h. The vitamin content was calculated by comparing the growth of the organism in the unknown samples with the growth of the organism in known standard concentrations by turbidimetric reading (Optical Density, OD, v / 575 nm) [35]. Vitamin B_6_ (sum of pyridoxine, pyridoxal, and pyridoxamine) was released from the sample by acid extraction, hydrolysis, and enzyme treatment and was determined by HPLC (reverse phase) using a fluorescence detector (Ex290 nm, Em390 nm; Agilent 1290 Infinity HPLC system, Agilent Technologies, PDA, Santa Clara, CA, USA). All samples were integrated using Chromeleon^Ⓡ^, and the vitamin B_6_ content was calculated by external calibration (standard curve) [36]. Vitamin B_9_ (folic acid) was released from the sample by extraction (autoclaving in acetate buffer) and mixed with growth medium, before the microorganism *Lactobacillus rhamnosus* (ATCC 7469) was added and incubated at 37°C for 20 h. The vitamin content was calculated by comparing the growth of the organism in the unknown samples with the growth of the organism in known standard concentrations, by turbidimetric reading (method based on the Swedish Nestlé AB's microbiological determination of folic acid in food, method nr. 71 C-2). Vitamin B_12_ (cobalamin) was released from the sample by extraction (autoclaving in acetate buffer) and mixed with growth medium, the microorganism *Lactobacillus delbruecki* (ATCC 4797) was added and incubated at 37°C for 22 h. The vitamin content was calculated by comparing the growth of the organism in known standard concentrations, by turbidimetric reading [35].

For determination of the Vitamin D_3_ (cholecalciferol) content, weighed sample material (0.2–1 g depending on the matrix) was mixed with 3 ml ethanol, 100 µl internal standard vitamin D_2_ (ergocalciferol, 0.5 µl/ml), one spatula tip of pyrogallol, one spatula tip of ascorbic acid, and 0.4 ml 37.5% KOH. The solution was mixed and saponified in a block-heater (100°C) for 20 min. After saponification, the mixture was cooled, before 1 ml distilled water and 3 ml n-hexane were added, mixed, and centrifuged. The hexane phase (upper layer) was transferred with a Pasteur-pipette into a 10 ml sample tube (vitamin D was totally extracted with 2 × 3 ml of n-hexane). The hexane phase was then washed with 2 ml distilled water. 1 ml iso-propanol was added before evaporated to dryness at ambient temperature under a N_2_ atmosphere. It was then added 0.3 ml of n-hexane and cleaned up using preparative HPLC (normal phase UV-detector, 254 nm; instrument conditions: column, Kromasil 100–3.5 µm SIL 150 × 4.6 mm; mobile phase: 15% (v/v) tetrahydrofuran in hexane; flow rate: 1 ml/min). In the preparative HPLC, the internal standard vitamin D_2_ and the vitamin D_3_ in the samples will eluate as one peak. The fraction containing D_2_ and D_3_ was pooled from 1 min before and after the peak. After collection of the peak, the fraction was evaporated to dryness on a block-heater under a N_2_ atmosphere, methanol was added, and the samples were shaken well and centrifuged if any precipitate was left in the tube. Vitamin D_2_ and D_3_ were separated and determined by HPLC (reverse phase) using an UV detector at 265 nm (HPLC LaChrom Merck HITACHI system, Tokyo, Japan; instrument conditions: Column, ACE 5 C18, 5 µm, 4.6 × 250 mm; mobile phase: 12 % (v/v) methanol and 6 % (v/v) chloroform in acetonitrile; flow rate: 1 ml/min). All samples were integrated using Chromeleon^Ⓡ^, and the content of vitamin D_3_ was calculated by internal standard D_2_. The method is based on the standards developed by the CEN [37].

For determination of the vitamin E (α-, β-, γ-, and δ-tocopherol and α-, β-, γ-, and δ-tocotrienol) content, weighed sample material (0.2–1 g depending on the matrix) was mixed with 4 ml ethanol, one spatula tip of pyrogallol, one spatula tip of ascorbic acid, 0.5 ml saturated EDTA, and 0.5 ml 20% KOH. The solution was saponified in a block-heater (100°C) for 20 min. After saponification, the mixture was cooled, and 1 ml distilled water and 2 ml n-hexane/ethyl acetate (80:20) were added. The solution was mixed, centrifuged, and the free vitamin E was extracted in the hexane face. The hexane phase (upper layer) was transferred with a Pasteur-pipette into a 10 ml sample tube (vitamin E was totally extracted with 3 × 2 ml of n-hexane/ethyl acetate). The hexane extract was then evaporated to dryness at ambient temperature under a N_2_ atmosphere and added an appropriate amount of n-hexane (depending on the matrix) for analysis by HPLC using a Fluorescence detector at EM 330 nm/EX 295 nm (HPLC UltiMate3000 system, Thermo Fisher Scientific, Waltham, MA, USA; instrument conditions: Pinnacle DB Silica 3 µm, 150 mm x 2.1 mm; mobile phase: hexane/ethyl acetate (80:20); flow rate: 0.3 ml/min). All samples were integrated using Chromeleon^Ⓡ^, and the content of α-, β-, γ-, and δ-tocopherol and α-, β-, γ-, and δ-tocotrienol were calculated by external calibration (standard curve). The method is based on the standards developed by the CEN [38].

## Determination of elements

The concentrations of elements (iodine (I), selenium (Se), zinc (Zn), iron (Fe), calcium (Ca), potassium (K), magnesium (Mg), phosphorus (P), and sodium (Na); arsenic (As), cadmium (Cd), mercury (Hg), and lead (Pb)) were determined by inductively coupled plasma-mass spectrometry (iCapQ ICP-MS, ThermoFisher Scientific, Waltham, MA, USA) equipped with an auto-sampler (FAST SC-4Q DX, Elemental Scientific, Omaha, NE, USA) after wet digestion in a microwave oven (UltraWave, Milestone, Sorisole, Italy), as described by Julshamn et al. (2007); [39]. Gold (Au) was added for the determination of mercury, in order to stabilise the element. The concentration of these elements was determined using an external standard curve in addition to an internal standard [40]. Three slightly different methods were applied: 1) for Ca, Na, K, Mg, and P, using scandium (Sc) as the internal standard, 2) for Zn, Fe, Se, As, Cd, Hg, and Pb, using rhodium (Rh) as the internal standard, and 3) for I, tellurium (Te) was used as the internal standard. For the determination of I, the sample preparation is a basic extraction with tetramethylammonium hydroxide (TMAH) before ICP-MS analysis. Data were collected and processed using the Agilent ChemStation, ICP-MS software (Agilent Technologies, Palo Alto, CA, USA). For methylmercury (MeHg) analyses, mercury species were analysed by GC isotope dilution ICPMS according to the method developed and described in detail by Valdersnes et al. (2012); [41]. The method involves spiking the tissue sample with Me^201^Hg, followed by decomposition with tetramethylammonium hydroxide, pH adjustment and derivatisation with sodium tetraethyl borate, and finally organic extraction of the derivatised MeHg in a hexane phase. Subsequently, the sample is analysed by GC-ICP-MS using an Agilent (Santa Clara, CA, USA) 6890 N gas chromatograph coupled to an Agilent 7500a ICP-MS instrument.

## Determination of organic pollutants

The organic pollutants analysed in this project were: dioxins (polychlorinated dibenzo-p-dioxins (PCDD) and polychlorinated dibenzofurans (PCDF), polychlorinated biphenyls (PCB; both dioxin-like PCBs and indicator PCBs), polybrominated diphenyl ethers (PBDEs), and polycyclic aromatic hydrocarbons (PAHs).

## Determination of dioxins, PCBs, and PBDEs

Samples were analysed for the 17 PCDD/F congeners which have been assigned Toxic Equivalency Factors (TEFs) by the World Health Organization (WHO; [40]): 2,3,7,8-TCDD; 1,2,3,7,8-PeCDD; 1,2,3,4,7,8-HxCDD; 1,2,3,6,7,8-HxCDD; 1,2,3,7,8,9-HxCDD; 1,2,3,4,6,7,8-HpCDD; OCDD; 2,3,7,8-TCDF; 1,2,3,7,8-PeCDF; 2,3,4,7,8-PeCDF; 1,2,3,4,7,8-HxCDF; 1,2,3,6,7,8-HxCDF; 1,2,3,7,8,9-HxCDF; 2,3,4,6,7,8-HxCDF; 1,2,3,4,6,7,8-HpCDF; 1,2,3,4,7,8,9-HpCDF, and OCDF. The dioxin-like PCBs analysed were also those assigned WHO-TEFs [42]: non-ortho PCBs; CB 77, 81, 126, and 169, and the mono-ortho PCBs; CB 105, 114, 118, 123, 156, 157, 167, and 189. Indicator PCBs (also called ICES-6 or PCB6) includes CB 28, 52, 101, 138, 153, and 180 (previously CB-118 was also included as an indicator PCB, as reported as ICES-7; now this congener is reported in sum DL-PCB). For PBDE, BDE 28, 47, 99, 100, 153, 154, and 183 (PBDE7) were analysed.

Sample material was mixed with hydro matrix and internal standards were added (13C labelled EDF-8999 for PCDD/F and EC-4937 for PCBs (Cambridge Isotope Laboratories, Andover, MA, USA)). For PBDEs, BDE 139 was used as the internal standard (Sigma-Aldrich, Andover, MA, USA). The analytes were extracted with hexane by an accelerated solvent extractor. The sample extracts were purified using three sequenced solid phase extraction columns (silica-, basic alumina-, and carbon column), on an automated PowerPrep system, (FMS, Waltham, MA, USA), as previously described [43]. PBDE, PCB-6, and mono-ortho-PCB were collected in one fraction, while PCDD/F and non-ortho PCB were collected in a second fraction. Both fractions were concentrated using Turbovap IITM (Zymark, USA). Remaining fat was removed in an external clean-up procedure by adding sulphuric acid (95-97%) to the extract.

PCDD/F and DL-PCB analysis was performed by high-resolution gas chromatography/high resolution mass spectrometry HRGC-HRMS (HRGC, Trace 2000 series; HRMS, DFS, Thermo Finnigan, Bremen, Germany), equipped with a fused silica capillary column (30 m x 0.25 mm i.d. and 0.25 µm film thickness, RTX-5SILMS, Restek, Bellefonte, USA). Recovery standards used were 13C labelled EDF-5999 for PCDD/F and EC-4979 for PCBs (Cambridge Isotope Laboratories, Andover, MA, USA).

PCB-6 and mono-ortho-PCBs were analysed by gas chromatography/tandem mass spectrometry (GC-MS/MS) (GC, 7890A; MS/MS, 7000B, Agilent Technologies, Germany), equipped with a fused silica capillary column (30 m x 0.25 mm i.d. and 0.25 µm film thickness, RTX-5SILMS, Restek, Bellefonte, USA).

PBDEs were analysed by negative chemical ionisation gas chromatography/mass spectrometry (GC, Trace 2000 series; MS, Trace DSQ, Thermo Finnigan, Bremen, Germany), equipped with a fused silica capillary column (30 m x 0.25 mm i.d. and 0.25 µm film thickness, RTX-5MS, Restek, Bellefonte, USA).

PCDD/F, DL-PCBs, and indicator PCBs were quantified according to the internal standard/isotope dilution method using congener-specific relative response factors (RRFs) determined from a three-point calibration curve according to the US EPA 1613 and 1668 method [44]. Final quantified PCCD/F and DL-PCB values are expressed as pg WHO-TEQ/g wet weight using the WHO-TEFs from 2005 according to EU legislation (EC, 2011) [45]. Quantification of PBDEs was performed by the internal standard approach using a seven-point congener specific calibration curve. Concentrations below the LOQ were reported as the LOQ (upper bound LOQ) to avoid underestimation of the risk.

## Determination of PAHs

The method for determination of PAHs quantifies 16 “European Food Safety Authorities (EFSA) PAHs”; benz(a)anthracene, benzo(a)pyrene, benzo(b)fluoranthene, benzo(c)fluorene, benzo(g,h,i)perylene, benzo(j)fluoranthene, benzo(k)fluoranthene, chrysene, cyclopenta(c,d)pyrene, dibenz(a,h)anthracene, indeno(1,2,3,-cd)pyrene, 5-methylchrysene, dibenzo(a,e)pyrene, dibenzo(a,h)pyrene, dibenzo(a,i)pyrene, and dibenzo(a,l)pyrene. The method is developed based on Varlet et al. (2007); [46], and Veyrand et al. (2007); [47].

Homogenised, freeze-dried samples of fillet or whole fish or homogenised samples of liver was weighed in (0.2–10 g, depending on matrix) and mixed with hydro matrix and silica gel and added an internal standard (US EPA 16 PAH Cocktail (13°C, 99%), CIL ES-4087), before extraction with solvents dichloromethane (DCM) and cyclohexane (1:3) on Accelerated Solvent Extractor (ASE 350, Dionex Corp.) at a temperature of 100°C and a pressure of 1500 psi in two cycles. Most of the lipids were removed with silica gel in the ASE cell. The extracts were concentrated by evaporation with nitrogen gas on a Turbo Vap (Turbo Vap II, Zymark) and further purified on an automated solid phase extractor (Aspec GX-374 Gilson) using SPE-column (ENVI Chrom P, Superclean, 250 mg/3 ml Supelco, Sigma Aldrich). The solvent was then changed from cyclohexane to isooctane and the samples were concentrated further to 50 µl and added recovery standard (3-Fluorchrysene, Chiron 1317.18-100-T) before being analysed with a GC-MS/MS instrument (GC: 7890A GC System; MS: 7000B Triple Quad) with autosampler (7693 Agilent Autosampler). The GC-column used was Select PAH, 15 m x 0.15 mm ID DF = 0.1 µm, Varian CP7461. With each sample series, a four-point calibration curve (15+1 EU PAH Cocktail, Chiron) was prepared and used for quantification, and software Agilent MassHunter Quantitative Analysis was applied to calculate the concentrations of the different analytes.

The limit of quantification (LOQ, ng) was 0.15 ng for most PAH analytes, except dibenzo(a,e)pyrene, dibenzo(a,h)pyrene, dibenzo(a,i)pyrene and dibenzo(a,l)pyrene for which LOQ was 0.75 ng (Table 1). The concentration of LOQ varied depending on the amount of sample weighed in (i.e. LOQ (ng/g wet weight) = LOQ (ng)/weight sample (g wet weight)). Measurement uncertainty (MU) was 30% for most analytes and 60% for the four dibenzo(a,x)pyrenes.

## Determination of microplastics

### Sample preparation: contamination avoidance

Tissue dissection was performed at the IMR laboratory, which is equipped with high efficiency ultra-low penetration HEPA filter with an efficiency of 99.995% for the most penetrating particle sizes. The laboratory has overpressure and the entrance a sluice with a sticky floor mat to avoid dust entry. It is entered with dedicated low-abrasion shoes and cotton laboratory coats. Clothing with loosely weaved artificial polymer fibres are avoided, and either no gloves or Nitrile gloves are worn. Wherever possible, non-plastic equipment is employed. Samples are handled under a laminar flow bench (Class II biological safety, Thermo Scientific SAFE 2020, LAF). Tissue samples are prepared with parallel procedural controls, i.e. at least duplicates of open glass jars of filtered 100 ml MilliQ water placed in the working area in the laboratory and in the LAF bench each working day, used to evaluate possible sample contamination from airborne plastic. Glassware used for sample preparation and analyses were pre-burned at 500°C to remove plastics contamination. Solutions for analyses were pre-filtered through fiberglass filters of a smaller pore size than the size of the analytes. Additionally, procedural blanks were run together with the processed samples following the same treatment steps, such as mincing and digestion, in order to estimate contamination through the reagents and instruments.

### Methods used for plastic analysis: microplastic extraction

The aim of the method was to extract microplastics from the investigated samples quantitatively and to apply a purification step prior to the analysis. The main interferents for a reliable quantification are organic matter, a complex mixture of proteins, and natural esters of glycerol, as well as various fatty acids which may trap and aggregate microplastics. These factors can reduce the efficiency of the extraction process, as well as interfere with the quantification process, causing an increase in the background signal and thus negatively influence the signal-to noise rate. As a starting point for the method implementation, a multi-step sequence of dispersants, enzymes, and oxidising treatments were tested in order to obtain optimal sample preparation and removal of interferents. The amount and type of enzymes need to be adjusted to the chemical composition of each sample type. Criteria for the method's performance evaluation are a) clogging and b) duration of the filtration step and signal-to noise ratio at quantification. For all matrices, optimisation of the purification steps is performed to minimise organic matter in the chemical identification of polymers by micro Fourier-Transform Infrared Microscopy (µFTIR; Agilent Cary 620/670; Agilent, Santa Clara, CA, USA) and pyrolysis-gas chromatography-mass spectrometry Orbitrap (py-GC/MS-Orbi, Thermo Fisher Scientific, Waltham, Massachusetts, USA). Recovery tests for polymers during treatments were performed to ensure that microplastics were not degraded or lost during the treatment conditions. Several recent publications have pinpointed that strong oxidising or alkali conditions agents, under high concentrations, high temperatures > 60˚C, and long incubation times > 48 h irreversibly damage some polymers. Therefore, the temperatures were limited to 50˚C and incubation time to 36 h. In general, an example protocol can be used as a starting point for extraction optimisation. In correct technical terms, no extraction is performed, but rather a dissolution of the natural organic matter, leaving the microplastics as intact as possible. Following is an example protocol for microplastic extraction:

1. Tissue may be ground by a plastic free meat mincer to increase chemical accessibility or to prepare homogeneous pooled samples without fine-cutting microplastics.
2. 20–100 g of tissue (wet weight) on glass crucible (D4, volume 125 ml, BRAND^Ⓡ^ filter crucible, Sigma-Aldrich Norway AS, Oslo, Norway).
3. +70 ml of Tween 20 (5%, v/v).
4. Sonicate for 1 min.
5. Incubate under movement at 50°C for 3 h.
6. Filtrate.
7. Rinse with water.
8. 90 ml protease and glycine buffer (0,1 M, pH 9) mixture (1:20); 48 h, 50°C.
9. Rinse with water.
10. In case of high fat/chitin content, consider adding a lipase and/or chitinase step.
11. 50 ml H_2_O_2_ (30%); 50°C, 36 h.
12. Wash. Consider repetition.
13. In case of high fat samples, consider polysorbate (Tween) 20 (5–10% in KOH 10% solution) 1:20, 24 h. Current method development points towards that protocols with KOH and detergent, combined with oxidative treatment but without enzymes, may prove a suitable and cheaper alternative for some tissues, such as fish fillets.

### Identification of microplastic through FTIR microscopy

FTIR is a type of vibrational spectroscopy. Different wavelengths are transmitted/reflected to a different extent by different polymers and can be measured as spectra with typical peaks or “fingerprint areas”, which can then be used to chemically identify plastic types, by comparison with libraries. We perform FTIR in two ways depending on particle size. A qualitative analysis of selected potential plastic particles over 500 µm is performed by Attenuated Total Reflectance FTIR (ATR-FTIR), while a quantitative analysis of microplastics from 10 to 500 µm is performed using FTIR microscopy. Due to the analysis of chemical identity through the transmission/reflection of infrared light, microplastics containing large amounts of carbon black, such as in car tires, will be detected to a lower extent by FTIR analysis.

### ATR-FTIR

Very few particles in seafood samples are larger than 500 µm. The particles are picked using tweezers, measured, weighed, and analysed using an ATR (GladiATR, Agilent, Santa Clara, CA, USA). If possible, three spectra are acquired for each particle. The obtained infrared spectra are compared to an openly available spectral library (https://simpleplastics.eu/download.html) and our own growing collection of spectra. Identification is accepted if the similarity score is more than 70%. If the match is between 60 and 70%, expert judgement of the spectra is applied to approve or reject the results. Below 60% the results are rejected.

### µFTIR imaging

Our µFTIR (Agilent Cary 620 FTIR microscope coupled to a Cary 670 FTIR spectrometer) system is equipped with a liquid nitrogen cooled 128 × 128 Focal Plane Array (FPA) detector, allowing for imaging of 128 × 128 pixels in a single measurement, a MIR Source with a spectral range of 9000–20/cm, purged enclosure, 15x IR/Vis reflective objective (NA 0.62, WD: 21 mm), 4x Vis glass objective (NA 0.2, WD 38 mm), motorised sample stage and 0.1 × 0.1 MCT (mercury, cadmium, telluride). Extracted environmental samples are filtered onto anodic aluminium oxide ceramic filters (Whatman^Ⓡ^ Anodisc inorganic filter membrane; Sigma-Aldrich) and imaged. Each pixel is imaged for the whole spectrometric range. Simultaneous optical images allow for the determination of the size of the particles in two dimensions. Usually, those two dimensions are the larger dimensions due to the filtration process. Automatic image processing smooths the edges of the determined microplastics and assigns a false colour coding for chemical identity, including polymer groups, to the microplastics. These data are statistically analysed according to number of particles per size and polymer group. With this system, both polymers and particle size distribution of an extracted sample can be determined, at least down to 10 µm. For dataset analysis, data was processed by siMPLE (Systematic Identification of MicroPLastics in the Environment [48] and spectra were compared to libraries from Bio-Rad and Agilent, the Alfred-Wegener Institute Helgoland (ref. Gunnar Gerdts, Sebastian Primpke) and IMR's own additions. Because the analysis method is non-destructive, the same samples can be analysed by py-GC/MS, subsequently, providing information about the total mass per polymer group in the same sample, and adding the possibility to measure microplastics below the size class of 10 µm, if there is enough mass to exceed the detection limit.

### Thermal degradation analysis - Py-GC/MS-Orbi

Pyrolysis gas Chromatography mass Spectrometry (Pyr-GC/MS-Orbi) is a thermal decomposition of materials at elevated temperatures in a low-oxygen atmosphere, avoiding burning. Large molecules break at their weakest bonds, producing smaller, more volatile fragments. These fragments can be separated by gas chromatography and detected by a mass spectrometer. Our specific mass spectrometer is a high-resolution instrument with better selectivity than a single quadrupole, also suited for screening [49]. The data can either be used as fingerprints to identify material, or to identify individual fragments to obtain structural information. The obtained pyrograms, with peaks of ions appearing at different retention times, are compared with a customised database and cross-checked with literature to identify the chemical composition of the material using recommendations and selecting criteria from Fischer and Scholz-Böttcher (2017; [50]) and Gomiero et al. (2019; [51]) and our growing experience. Standard curves with known concentrations are used to calculate the concentrations of materials present in the sample. Differently to FTIR, Py-GC/MS-Orbi is a destructive method that irreversibly degrades the polymers and does not produce an image of the material, but it provides the mass down to the ng range of the identified polymers independent of the particle size and is not limited by the transmissibility of light of the material. The two methods FTIR and py-GC/MS-Orbi are therefore complementary and increase the information gained from an extracted sample. Our Orbitrap mass spectrometer Thermo QExactiver is coupled with Frontiers Multi-Shot Pyrolizer EGA/PY-3030D with an auto-shot sampler.

## Declaration of Competing Interest

The authors declare that they have no known competing financial interests or personal relationships that could have appeared to influence the work reported in this paper.

## Appendix Supplementary materials

Image, application 1

Image, application 2

Image, application 3

Image, application 4

Supplementary material: The sampling protocols are described in protocols 1-5 in the supplementary materials. Image, application 5
