# Corrigendum to ‘‘Sampling protocol for the determination of nutrients and contaminants in fish and other seafood – The EAF-Nansen programme” [MethodsX 7 (2020) 101063]

**DOI:** 10.1016/j.mex.2021.101363

**Published:** 2021-04-21

**Authors:** Amalie Moxness Reksten, Annbjørg Bøkevoll, Sylvia Frantzen, Anne-Katrine Lundebye, Tanja Kögel, Kjersti Kolås, Inger Aakre, Marian Kjellevold

**Affiliations:** Institute of Marine Research, P.O. Box 2029 Nordnes, Bergen 5817, Norway

The authors regret to inform that part of the methodology related to the description of the determination of dioxins, PCBs, and PBDEs in the published paper is outdated. This also relates to the description of some of the certified control materials (CRM) and internal control materials (Tables 2 and 3) used for the determination of these congeners. This method is complex and in constant development; the corrected and updated description of this method and the corrected tables for these congeners appear below.

**Table 2:** Overview of certified reference materials (CRM).

Analyte	Reference materials	Certified value ± U= 2*u	Analysed value ± U= 2*u	Unit	Mean Accuracy (%)
PBDE 196	Not part of test				
PBDE 197	Not part of test				
PBDE 206	Not part of test				
PBDE 207	Not part of test				
PBDE 209	FHI 2015 Interlab. test C	22 ± 20	31 ± 2.8	pg/g	150

**Table 3:** Overview of internal control materials.

Analyte	Control material	Analysed value ± U= 2*u	2RSD (%)	Unit
PBDE 196	Freeze-dried salmon (2015-1388), spiked	2.61 ± 0.24	9	ng/g
PBDE 197	Freeze-dried salmon (2015-1388), spiked	0.25 ± 0.01	5	ng/g
PBDE 206	Freeze-dried salmon (2015-1388), spiked	2.46 ± 0.08	4	ng/g
PBDE 207	Freeze-dried salmon (2015-1388), spiked	0.51 ± 0.02	5	ng/g
PBDE 209	Freeze-dried salmon (2015-1388), spiked	1.08 ± 0.1	10	ng/g

The authors would like to apologise for any inconvenience caused.

## Determination of dioxins, PCBs, and PBDEs

Samples were analysed for the 17 PCDD/F congeners which have been assigned Toxic Equivalency Factors (TEFs) by the World Health Organization (WHO; [40]): 2,3,7,8-TCDD; 1,2,3,7,8-PeCDD; 1,2,3,4,7,8-HxCDD; 1,2,3,6,7,8-HxCDD; 1,2,3,7,8,9-HxCDD; 1,2,3,4,6,7,8-HpCDD; OCDD; 2,3,7,8-TCDF; 1,2,3,7,8-PeCDF; 2,3,4,7,8-PeCDF; 1,2,3,4,7,8-HxCDF; 1,2,3,6,7,8-HxCDF; 1,2,3,7,8,9-HxCDF; 2,3,4,6,7,8-HxCDF; 1,2,3,4,6,7,8-HpCDF; 1,2,3,4,7,8,9-HpCDF, and OCDF. The dioxin-like PCBs analysed were also those assigned WHO-TEFs [42]: non-ortho PCBs; CB 77, 81, 126, and 169, and the mono-ortho PCBs; CB 105, 114, 118, 123, 156, 157, 167, and 189. Indicator PCBs (also called ICES-6 or PCB6) include the non-dioxin-like CB 28, 52, 101, 138, 153, and 180 (previously CB-118 was also included as an indicator PCB, as reported as ICES-7; now this congener is reported in sum DL-PCB). An extra non-dioxin-like PCB, CB-31, was included from 2020. For PBDEs, the tri-hepta BDEs 28, 47, 49, 66, 99, 100, 119, 138 153, 154, and 183 were analysed, and the tri-hepta BDEs 35, 71, 75, 85, and 118 were reported from 2020. The octa-deka PBDEs BDE 196, 197, 206, 207 and 209, were also reported from 2020. Often PBDE results are presented as sum PBDE7 (BDE 28, 47, 99, 100, 153, 154, and 183).

Sample material was mixed with hydro matrix and internal standards were added (13C labelled EDF-8999 for PCDD/F, EC-5379 and EC-4937 for PCBs, EO-5100-10X, EO-5035, EO-5161, EO-5337, EO-5240, EO-5241, and EO-5003 for PBDEs (Cambridge Isotope Laboratories, Andover, MA, USA)). The analytes were extracted with hexane by an accelerated solvent extractor (ASE 350®, Dionex Corporation, Sunnyvale, CA, USA) under elevated pressure and temperature (100°C, 1500 psi) and concentrated using Turbovap II (Zymark/discontinued, Caliper Life Sciences, Mountain View, CA, USA). The sample extracts were purified using high performance clean-up columns (silver nitrate-, sulfuric acid/silica-, carbon- and alumina column), on an automated GO-6HT system (DSP-Systems, Ede, Netherlands). PBDEs, ndl-PCBs, and mono-ortho-PCBs were collected in one fraction, whereas PCDD/Fs and non-ortho PCBs were collected in a second fraction. Furthermore, solvent exchange and adding of recovery standards (S-5117-ASS-NN and S-5118-ASS-NN, Chiron, Trondheim, Norway) were executed by automated liquid handling (Microlab Starlet, Hamilton Nordic AB, Kista, Sweden).

PCDD/F and non-ortho-PCB analysis was performed by high-resolution gas chromatography/high resolution mass spectrometry (HRGC-HRMS) (HRGC, Trace 2000 series; HRMS, DFS, ThermoFisher Scientific, Waltham, MA, USA), equipped with a fused silica capillary column (30 m x 0.25 mm I.D. and 0.25 µm film thickness, Rxi-5SIL MS, Restek, Bellefonte, USA).

Ndl-PCBs, mono-ortho-PCBs and tri-hepta PBDEs were analysed by gas chromatography/tandem mass spectrometry (GC-MS/MS) (GC, 7890A; MS/MS, 7000B/C, Agilent Technologies, Santa Clara, CA, USA), equipped with a low bleed proprietary phased column (30 m x 0.25 mm I.D. and 0.25 µm film thickness, Agilent J&W, Santa Clara, CA, USA).

Octa-deka PBDEs were analysed by negative chemical ionisation GC/MS (GC, Trace 1310; MS, DSQ, ThermoFisher Scientific, Waltham, MA, USA), equipped with a fused silica capillary column (15 m x 0.25 mm I.D. and 0.1 µm film thickness, RTX-1614, Restek, Bellefonte, USA).

Quantification of congeners was performed according to the isotope dilution/internal standard method using relative response factors (RRs) or response factors (RFs) determined from calibration curves. For quantification of PCDD/Fs and non-ortho PCBs, a five-point calibration curve was used, according to the US EPA 1613 and 1668 method [44]. Mono-ortho PCBs, ndl-PCBs, and tri-hepta PBDEs were quantified using eight-point calibration curves. Okta-deka PBDEs were quantified using five-point calibration curves weighted 1/*S*^2^.

Final quantified PCCD/F and DL-PCB values are expressed as pg WHO-TEQ/g wet weight using the WHO-TEFs from 2005 according to EU legislation (EC, 2011) [45]. Concentrations below the LOQ were reported as the LOQ (upper bound LOQ) to avoid underestimation of the risk.

