# Supplementary material for: Sampling protocol for the determination of nutrients and contaminants in fish and other seafood – The EAF-Nansen Programme
Source: MethodsX. 2020 Sep 12;7:101063. doi: 10.1016/j.mex.2020.101063 (PMC7502570; doi:10.1016/j.mex.2020.101063)
Supplement: Supplementary file 1 [file mmc1.docx]

Sampling Protocol 1: General information

**Background**

Samples of fish and other seafood collected on EAF Nansen cruises will be analysed for a range of nutrients in order to document the importance of these fish species to local food and nutrition security. In addition, they will be analysed for contaminants in order to ensure food safety. It is important to assess whether marine pollution affects food safety and the marine environment in general.

The samples will therefore be analysed for many different nutrients and contaminants, with advanced analytical methods at the IMR’s laboratories in Bergen, Norway. Sampling the fish, homogenising and freeze-drying the sample material will be done on board the ship. These are essential steps of the analysis which can significantly affect the results, and it is therefore very important that this is done properly. It is also important that as much relevant information as possible about the fish is registered.

Ideally and preferably, the fish is processed immediately following sampling, as too much freezing and thawing can affect the levels of some nutrients. However, if this is not possible, the fish can be frozen after sampling, then be partially thawed and processed later.

This document is meant primarily as background reading material. For a more hands-on description of the work to be done on board the vessel, see separate protocols for small fish and large fish, respectively.

**Definitions**

Sampling position: The geographical area where the fish to be sampled were caught, given by geographical coordinates. The coordinates may represent the place where a trawl haul was pulled in. Sometimes in the open sea a position may represent a relatively large area, e.g. an area with a radius of 2 km.

Sample: The object to be described through the analysis – for example this may consist of muscle tissue from one fish or a pool of 25 whole fish.

Fillet: Soft tissue between skin and bone, consisting of skeleton musculature and fatty tissue. This is the part of the fish that is most often consumed. It contains both brown and white muscle. A fillet can be with or without skin. It is important that as much as possible of the fillet is sampled from each fish.

Fillet with skin and bone: Whole fish from which the internal organs have been removed and the head and tail have been cut off.

Composite or pooled sample: A sample composed of tissue from several fish. The objective here is to ensure enough material for all the analyses and that each sample is representative of the sampled population.

Homogenising: Grinding and mixing a sample until the composition is identical throughout.

Homogenous sample: If the sample is split into several parts, the composition of each part will be identical.

Sub-sample: A representative part of a sample, separated out for further analysis.

Secondary sampling: When there are large composite samples, an sub-sample of the composite sample is taken for further analysis. Secondary sampling should take place according to regular routines to ensure representativeness.

Freeze-drying: Removing water from the sample using vacuum and low temperature in order to make a dry sample which is durable, and to determine water contents of the sample. Many analyses apply dried samples. Freeze-drying is done with a specialised apparatus, a freeze-dryer.

**Sampling**

The aim is to sample and analyse fish that are important in terms of volume and as a food source for people in the region. It is important that we sample edible tissues of the fish, i.e. the fillet of large fish and fillet with skin and bone or whole fish of small fish. However, in order to assess pollution levels, we also want a tissue that is particularly sensitive to pollution. We therefore also take samples of liver of the large fish, although this is not directly a food safety issue.

For each species, we want samples from two-three different sampling positions on each cruise leg. The positions should be well separated geographically.

The fish samples are separated into two main types: "small" fish and "large" fish. Examples of fish species in the small and large categories as defined here are given in Table *1*.

**Table 1**. Overview of possible species in the small fish and large fish categories, tissue to be analysed, type of sample and the number of samples to be taken.

| Species of interest | Tissue | Sample | Number of samples at each position |
| --- | --- | --- | --- |
| Small fish |  |  |  |
| Pilchard, *Sardina pilchardus* | Whole fish | Composite of 25 fish | 3 samples |
|  | Fillet with skin and bone | Composite of 25 fish | 3 samples |
| Anchovy, *Engraulis encrasicolus* | Whole fish | Composite of 25 fish | 3 samples |
|  | Fillet with skin and bone | Composite of 25 fish | 3 samples |
| Round sardinella, *Sardinella aurita* | Whole fish | Composite of 25 fish | 3 samples |
|  | Fillet with skin and bone | Composite of 25 fish | 3 samples |
| Flat sardinella, *Sardinella maderensis* | Whole fish | Composite of 25 fish | 3 samples |
|  | Fillet with skin and bone | Composite of 25 fish | 3 samples |
| Large fish |  |  |  |
| Horse mackerel, *Trachurus trachurus* | Fillet | Individual | 25 fish |
|  | Liver | Individual | 15 fish |
| Atlantic chub mackerel, *Scomber colias* | Fillet | Individual | 25 fish |
|  | Liver | Individual | 15 fish |
| Axillary seabream, *Pagellus acarne* | Fillet | Individual | 25 fish |
|  | Liver | Individual | 15 fish |

**Freeze-drying**

After homogenising the sample, weigh in the necessary amount to be freeze-dried in a tared and labelled container. Write down the weight of the container and the weight of the wet sample. The sample should not be thicker than 2 cm. Put on a lid and freeze the sample. The sample must be frozen before it is inserted into the freeze-drier. When it is ready for freeze-drying, remove the lid, and put the sample inside the freeze drier and the process is started, see the user instruction for freeze drier, below. After 24 hours the plate temperature is changed to +25°C, see instruction for freeze drier.

The freeze-dryer should run for 72 hours or more. When the freeze-drying has finished the sample must be weighed and remember to write down the weight (g). After weighing, the dried sample is homogenised once more. Freeze dried samples may draw humidity from the air. It is therefore important that the sample is placed in exicator cabinet if it is not weighed immediately after freeze-drying.

**Practical remarks**

The freeze drier is maintained by wiping off shelves, door and capacitor and keeping them clean. The oil is changed after 6-12 months, depending on the colour of the oil. The oil should have a clear colour. If changing the oil, see user guidance for the vacuum pump.

**After freeze-drying**

After the samples have been freeze-dried, check that they are completely dry by breaking them in half. They should have a biscuit-like texture. Then homogenise to a fine powder and transfer to one or two pre-labelled 50 ml tubes. Vacuum-pack the tubes from one station and species, label the bag and store at -20°C. See separate instruction for the vacuum sealer.

## **User guide for the freeze drier**

### Start freeze drier

1. Turn on the two main switches on the left side of the freeze drier
2. Place baffle in the correct direction (se illustration)
3. Put the glass lid over the collector and plug the tube.
4. Check the oil level. It should be between min. and max.
5. Push "Man". Wait until the temperature of the collector is ca. -50°C. When all the lamps on the temperature curve are alight, the freeze drier is ready.
6. Check that the black handle on the vacuum pump is pointing straight upwards
7. Put frozen samples in the shelves, making sure that the wires in the inner part of the shelves and the temperature sensors are not in a squeeze
8. Close the door. Turn the handle "Vac Release" to position close.
9. Push "VACUUM"
10. Push "SET TEMP" and lower the shelf temperature using the arrow button to run -20°C
11. After 24 hours, change "Set temp" (shelf temperature) to +25°C using the arrow buttons.

### Stop freeze drier

1. Turn off "VACUUM"
2. Turn the handle "Vac Release" to position "open"
3. Wait until the hissing sound stops before opening the door. Do not use power
4. Take out the samples
5. Unplug the tube, and check that the tube is hanging into the bucket. The water will run into the bucket
6. Turn off "Man"
7. Push "Defrost". It turns off automatically
8. Lift off the glass lid
9. Pull up baffle, and take out the ice lumps
10. Wipe off the rest of the moisture in the collector with paper
11. Wipe off the door and around the collector with glass wipe or methyl ethanol. Wipe off the shelves when needed
12. Leave the door open, the glass lid is set aside so the freeze drier can air off. Turn off the main switches.
