# Supplementary material for: Sampling protocol for the determination of nutrients and contaminants in fish and other seafood – The EAF-Nansen Programme
Source: MethodsX. 2020 Sep 12;7:101063. doi: 10.1016/j.mex.2020.101063 (PMC7502570; doi:10.1016/j.mex.2020.101063)
Supplement: Supplementary file 2 [file mmc2.docx]

Sampling protocol 2: Small fish

Sample 3 x composite samples of 25 whole fish and 3 x composite samples of 25 fillets with skin, bones, and viscera intact (a total of 150 fish). A composite sample should contain at least 25 individuals or 120 g wet sample material. Try to avoid direct sunlight on the samples for longer time periods. If necessary, cover with aluminium foil. Keep the fish as cold as possible - use the refrigerator if necessary.

**Procedure:**

1. Collect 150 fish in a basket. Print out a copy of the “Station form” for the correct station/trawl from the software NANSIS (see “Saving data during and after a survey”). Additionally, have a working sheet (“Trawl form – Small fish”) ready before staring any fish handling. Note the correct journal number on the form ("2020-xxx"). Each journal number corresponds to the species sampled from each trawl.
2. Weigh and write down the total weight of one composite sample consisting of 25 fish. If you don’t get average length from the catch (NANSIS), you will need to measure the length of 25 fish. Write down the length of each of the 25 fish (on the reverse side of the sheet). Write the mean weight and length in the trawl form.
3. You will need:
   - Cutting board
   - Filleting knife
   - Six tubs
4. Count 25 fish in each tub
   - Weigh the 25 fish in each tub.
   - If the fish have large scales, these should be scraped off as well as you are able to.
   - For three of the tubs, prepare fillet samples: open the fish from the gut opening, take out all the viscera (inner organs). Cut off the head and the tail. Wash the fish to remove blood and remains of the viscera.
   - For the next three tubs you keep the fish as is = whole fish sample.
   - Wash all the equipment with soap and a brush and clean the workspace when you are done.
5. When you have finished filleting all the fish, take out the following:
   - Food processor
   - 6 x 50 ml tubes for wet samples. Label with pre-printed labels, and sort by increasing number. Be aware that there are 3 tubes for whole fish (samples 1-3), and 3 tubes for fillets (samples 4-6).
   - 6 x salad trays and lids.
   - Spatula (baking type)
   - Spatula (lab type) or spoon
   - Permanent marker
6. Homogenisation:
   - The fish from each tub are now to be homogenised in the food processor. Put the 25 whole fish or fish fillets in the food processor and run the food processor until you have a homogenous paste. Fill one 50 ml tube with paste (check that you have the right number on the tube, it should correspond to the number on the fish and marked with whole fish = ”hel fisk”, or fillet = “filet”).
   - Label salad trays with pre-printed labels. Add about 120 g paste to a salad tray, filling to no more than 2 cm height.
   - Repeat this for all the fish samples (all 6 tubs).
   - Gather all 50 ml tubes with wet sample in one bag, label well with species, station no., date and journal no. (see tubes). Put the bag in the freezer.
7. Weigh each salad tray with contents on the two-decimal scale. Note wet weight in the form per sample. Freeze the samples for at least 12 hours at -20⁰C or lower.
8. Freeze-dry the samples for 72 hours. Remember to turn up the temperature after 24 hours. See a separate instruction on the freeze drier. Remember to take off the lid before freeze drying.
9. Check that the samples are completely dry by breaking a sample in two, and check that it is dry inside (biscuit consistency). As soon as the samples are done freeze-drying, weigh each sample with tray and lid on the two-decimal scale. Note the weight per sample in the form.
   - If you don't have time to weigh the samples immediately, put them in the exicator cabinet. Check that the silica gel in the bottom are orange. If it is blue you need to change it and dry the old ones in a drying cabinet. You will find new silica gel in the chemical closet.
10. Homogenisation after freeze-drying. You will need:
    1. Use the new blender for this (the black hand blender is not good enough).
    2. Funnel made of wet paper sheets (prepare using wet paper/photo paper and tape – should fit into the 50 ml tube) or plastic funnel.
    3. Up to 6 x 50 ml tubes. Label 1 tube for each pooled sample. Sort in a rack in increasing order.
    4. A brush to clean out the dust between samples.
    5. A sieve to sift out remaining whole scales.

Break the freeze-dried sample into the blender bowl. Mix in the blender until you have a homogeneous powder. If there are a lot of visible fish scales that have not been homogenised, they can be removed by sifting the powder through a sieve. Add the powder to a 50 ml tube. NB! Check that the number and letter on the tube corresponds with the number and letter on the tray. Repeat for all samples. Clean the workspace and the equipment when you are finished.

1. Vacuum-packing the samples

When all the samples have been put in tubes, they must be vacuum packed and put in the freezer. How to use the vacuum machine: Put the tubes into a vacuum bag, lid against lid. Avoid filling the bag completely. There should be app. 7 cm left. Put the end of the bag inside the machine (the end of the vacuum bag should touch the black pegs inside the machine, but not cover the vacuum hole). Make sure the lid is closed properly on both sides. Push the “Vacuum and seal” button. Wait until finished. Count the tubes and weigh the bag. Put the bag in a marked box for dry samples in the freezer.

1. Make sure to enter all information into the trawl form and bring all the work sheets back home.
2. Fill out the form “Overview of samples” for each fish species sampled. See the protocol for “Saving data during and after a survey”.
