# Supplementary material for: Sampling protocol for the determination of nutrients and contaminants in fish and other seafood – The EAF-Nansen Programme
Source: MethodsX. 2020 Sep 12;7:101063. doi: 10.1016/j.mex.2020.101063 (PMC7502570; doi:10.1016/j.mex.2020.101063)
Supplement: Supplementary file 3 [file mmc3.docx]

Sampling protocol 3: Large fish

Each fish must be at least 25 cm long to obtain enough sample material for the planned analyses. From each trawl, aim to sample 25 individual fish. Try to avoid direct sunlight on the samples over a long time period. Cover with aluminum foil if necessary. Keep the fish as cold as possible - use the refrigerator if necessary.

**Procedure:**

1. Collect 25 fish in a basket. Print out a copy of the “Station form” for the correct station/trawl from the software NANSIS (see “Saving data during and after a survey”). Additionally, have a working sheet (“Trawl form – Large fish”) ready before staring any fish handling. Note the correct journal number on the form ("2020-xxx"). Each journal number corresponds to each species sampled from the trawl.
2. Weigh and measure the fish. Label each fish with a number (1-25). Write individual weight and length in the Trawl form.
3. You will need:

- 15 x 12.5 ml Nunc tray for liver samples. Label them with pre-printed labels, and sort by number.
- 15 x Lids for the 12,5 ml Nunc trays.
- Scalpel with scalpel blade
- Tweezer
- Cutting board
- Filleting knife
- Cover a shelf in the refrigerator with aluminium foil

1. For each fish, do the following:
   - Open the fish from the gut opening, take out the internal organs. Write the gender in the form. Cut loose the liver from the first 15 fish and put it in the correct Nunc tray (the number of the fish should correspond to the number on the tray).
   - Fillet the fish and remove the skin from the fillet. Remove visible bones. NOTE: it is important to include as much of the muscle tissue as possible. Make sure to include the red/brown tissue along the skin. Put the fillet with the number-note on, in the fridge on the aluminium foil.
   - Wipe off the cutting board, scalpel and tweezer between each fish using a paper towel.
   - Wash all the equipment with a soap and a brush and clean the workspace when you are finished.
   - Put all the liver samples in a bag labelled well with species, station no., date, journal no. and "liver samples". Put in the freezer.
2. Once you have finished filleting all the fish, take out the following:

- Food processor
- 25 x 50 ml tubes for wet samples, label them with pre-printed labels. Sort in a rack.
- 30 x salad trays and lids
- Baking spatula
- Laboratory spatula or spoon.

1. Homogenisation:

All the fillets from each fish is now to be homogenised in the food processor. Put the fish inside and run the blender until you have a homogenous paste.

- Fill a 50 ml tube with at least 30 ml paste (check that you have the right number on the tube – should correspond to the fish number).
- Label salad trays with pre-printed labels.
- Add ca. 120 g paste to a salad tray.
- Repeat this for all the fillets.
- Gather all 50 ml tubes with wet sample in one bag, label well with species, station no., date and journal no. (see tubes). Put in the freezer.

1. Make 5 pooled samples of the 25 fillets:
   - Take 20 g homogenized paste from each of the 5 first salad trays, homogenise, and add to a new tray, labelled "fish 1-5"
   - Repeat for the next 5 fish and add to a new tray labelled "fish 6-10".
   - Repeat for the next 5 fish and add to a new tray labelled "fish 11-15".
   - Repeat for the next 5 fish and add to a new tray labelled "fish 16-20".
2. Repeat for the next 5 fish and add to a new tray labelled "fish 21-25". Weigh all salad trays with lid and contents on the two-decimal scale. Note the wet weight in the form per sample. Freeze the samples for at least 12 hours (ensure thorough freezing of the samples) at -20⁰C.
3. Freeze-dry the samples. Remember to turn up the temperature after 24 hours. See a separate instruction for the freeze-dryer. Remember to take off the lid of the tray before freeze-drying.
4. Check that the samples are entirely dry by breaking a sample in two, and check that it is dry inside (biscuit consistency). As soon as the samples are done freeze-drying, put on new lids and weigh the samples on the two-decimal scale. Note the weight per sample in the form. If you don't have time to weigh the samples immediately, put them in the exicator cabinet. Check that the silica gel in the bottom is orange. If it is blue you have to change it and dry the old ones in a drying cabinet.
5. Homogenisation after freeze-drying. You will need:

- Hand blender (little black one)
- Funnel made of wet paper sheets (prepare using wet paper/photo paper and tape – should fit into the 50 ml tube) or plastic funnel.
- Up to 65 x 50 ml tubes. Label 1 or 2 tubes for each fish and for each pooled sample. Sort in a rack in increasing order.
- A brush to clean out the dust between samples.

1. Break the freeze-dried sample into the hand blender bowl. Mix using the hand blender until you have a homogeneous powder. Put the powder in 50 ml tubes. NB! Check that the number on the tube corresponds with the number on the tray. Repeat for all samples. Clean the workspace and the equipment when you are finished.

The lids for the salad trays can be re-used.

1. Vacuum packing

When all the samples have been put in tubes, they must be vacuum packed and put in the freezer.

How to use the vacuum machine: Put the tubes into a vacuum bag, lid against lid. Avoid filling the bag completely. There should be app. 7 cm left. Put the end of the bag inside the machine (the end of the vacuum bag should touch the black pegs inside the machine, but not cover the vacuum hole). Make sure the lid is closed properly on both sides. Push the “Vacuum and seal” button. Wait until finished. Put the bag in the freezer.

1. Make sure to bring all the working sheets back home.
2. Fill out the form “Overview of samples” for each fish species sampled during the survey. See the protocol for “Saving data during and after a survey”.
