# Supplementary material for: Sampling protocol for the determination of nutrients and contaminants in fish and other seafood – The EAF-Nansen Programme
Source: MethodsX. 2020 Sep 12;7:101063. doi: 10.1016/j.mex.2020.101063 (PMC7502570; doi:10.1016/j.mex.2020.101063)
Supplement: Supplementary file 4 [file mmc4.docx]

Sampling protocol 4: Mesopelagic species

**Background**

The aim is to sample and analyse fish that are caught in the mesopelagic trawl. Mesopelagic fish are generally small (2-15 cm) and will be analysed as composite samples of whole species.

Experience from previous mesopelagic transects suggest that a mesopelagic trawl contains a mixture of many different species. In order to get high-quality data, the mesopelagic fish must be analysed species by species. Species determination before sampling is therefore crucial.

Collect samples of one or a few species that are dominant in each trawl, or if none are dominating, collect several species. Often there are few individuals of each species. A list of species sampled during the previous mesopelagic transects is presented in Table 1, and if these are in the catch, they are particularly interesting. However, if other species than the ones in the list are dominating, these should also be sampled as there are presently very little data on mesopelagic species.

Before you can start processing the fish, a fish taxonomist must identify them to correct species. Talk to the fish taxonomist who does the mesopelagic fish identification and inform him/her about your work.

**Table 1**: Overview of species of mesopelagic fish sampled during the mesopelagic transect in Las Palmas 2017.

| Species name in Latin | "Norwegian name" | English name |
| --- | --- | --- |
| *Argyropelecus aculeatus* | Pigghalet perlemorfisk | Longspine silver hatchetfish |
| *Chauliodus sloani* | Hoggormfisk | Manylight viperfish |
| *Diaphus bertelseni* | Bertelsens lysprikkfisk* | Bertelsen's lantern fish |
| *Diaphus dumerilii* | Lysprikkfisk *D. dumerlii* | Lantern fish |
| *Diaphus rafinesquii* | Hvitflekket lysprikkfisk | White-spotted lantern fish |
| *Diretmus argenteus* | Sølvdukatfisk* | Silver spinyfin |
| *Gonostoma denudatum* | Børstemunn* | Bristlemouth |
| *Macroramphosus gracilis* | Trompetfisk | Slender snipefish |
| *Margrethia obtusirostra* | Storhodet porthullfisk* | Bighead portholefish |
| *Maurolicus muelleri* | Laksesild | Silvery lightfish |
| *Myctophum selenops* | Wisners lysprikkfisk* | Wisner's lantern fish |
| *Nannobrachium lineatum* | Lyprikkfisk *N. lineatum* | Lantern fish |

*The Norwegian names are partly self-invented.

**Preparation of samples**

Mesopelagic fish are to be analysed as composite samples of whole fish. A composite sample should contain at least 25 fish or 120 g wet sample material. Because of the small size of mesopelagic fish, you may need more than 25 fish to get 120 g sample material.

If there are many of the same species in one trawl, take up to 3 parallel composite samples of the same species from the same trawl. One pooled sample is sufficient if there is a limited amount of fish. If, on the other hand, there is very little of each species, you take as much as you can get.

- For each sample, total weight (g) and the number of fish must be noted.
- For each sample, put all the fish into the food processor and homogenise. Make sure to run the food processor long enough to get a properly homogenised paste.
- Take out a wet sample: add approximately 20 g of the homogenised paste to a labelled 50 ml tube.
- Add the rest of the homogenized paste to a labelled salad tray. Fill the salad tray to no more than 2 cm height. Freeze-dry.
- Fill out the form for “Overview of samples” for each fish species sampled. See the protocol for “Saving data during and after a survey”.

**Freeze-drying, calculation of dry matter and water content, and vacuum-sealing**

This process is the same as for small and large fish. See respective protocols.

**Sampling of mesopelagic fish for analyses for microplastics**

- When working with samples for microplastics, do not wear fleece clothing. Do not use plastic gloves, but make sure your hands are clean.
- Samples to be analysed for microplastics must be handled as little as possible, due to danger of contamination. Therefore, samples of whole fish are to be taken directly from the catch and put into 150 ml glass jars (jam-type).
- Samples of about 100 g is required, but the contents of the jar must not touch the plastic cover. As a rule, the contents should be no less than 2 cm from the cover. Shut the lid once, do not open and close repeatedly, this may lead to plastic flaking.
- Ideally, we would like samples of individual species. However, identification is probably difficult to achieve directly from the catch since they should be handled as little as possible. Therefore, mixed samples are accepted. It would be good, though, if a light sorting could be done. This could be to separate for example crustaceans and fish. Or, if possible, separate all lanternfish (many species) from other fish types.
- Number of samples: Take as many as you can while you are at it.
- The samples should be frozen as soon as possible, making sure to keep the jars upright during storage.
