# Supplementary material for: Sampling protocol for the determination of nutrients and contaminants in fish and other seafood – The EAF-Nansen Programme
Source: MethodsX. 2020 Sep 12;7:101063. doi: 10.1016/j.mex.2020.101063 (PMC7502570; doi:10.1016/j.mex.2020.101063)
Supplement: Supplementary file 5 — Supplementary material: The sampling protocols are described in protocols 1-5 in the supplementary materials. [file mmc5.docx]

Sampling protocol 5: Microplastic analyses

**Background**

The Institute of Marine Research analyses seafood in order to establish baseline information of the microplastic content in whole fish species, fish fillet, and fish livers of the most important commercial seafood species. The best practice for microplastic analyses of seafood is to sample frozen, whole fish. The fish should be single packed into plastic bags and frozen as soon as possible. The single packing is necessary to ensure a shorter and more homogeneous thawing, and avoidance of contamination of the lower fish by run-off from entrails of the upper fish. Fish are thawed lying on the side, and if fillet is analysed separately from the rest of the fish, the lower fillet is excluded from analysis for the same reason. If the species in question have very small individuals, samples should be pooled to 100 g into food grade glass jars, frozen upright, on board, as soon as possible, and sent frozen inside these jars to the laboratory. The food grade jars of 150 ml should be pre-cleaned thrice with water and ethanol, filtered with a pore size of less than the microplastic to be investigated and of non-plastic material. Jars should be dried in a dust free environment and closed only once before adding the sample and once after adding the sample. The reason behind this is that the lock will be coated with plastic, and repetitive use of a lock with plastic coating will lead to plastic abrasion and contamination. Ideally, several such jars should be somewhat filled with pre-filtered water, such as with 30 ml in the dust free laboratory and frozen. They should be transported to the vessel frozen to avoid contact of the water with the lock. During processing of the catch, jars should be placed openly in the working area to sample dust controls that might contaminate the catch under the processing. After the work, jars should be closed, frozen, and sent frozen to the laboratory, together with the samples, as procedural controls to be analysed together with the samples.

To ensure all necessary data are provided (trawl location, species name, etc.), printed trawl forms are to be added in the bag with the frozen fish (and saved electronically on a computer, see “Saving data during and after a survey” for details).

**Procedure**:

1. Print out a copy of the “Station form” for the correct station/trawl from the software NANSIS (see “Saving data during and after a survey”). Additionally, have a working sheet (“Trawl form – Small/Large fish”) ready before staring any fish handling. Note the correct journal number on the form ("2020-xxx"). Each journal number corresponds to the species sampled from each trawl.
2. Collect 25 individuals of each species heavier than 100 g per fish, from each trawl. Fish can be bled, but should be whole (with head, tail, and viscera). The samples need to be of good quality, such as it could be sold as food. The individual’s size range should reflect what is usually caught, preferably with a good size distribution within that frame. The fish should be single packed, each in its own plastic bag before freezing. No contamination precautions or blank samples necessary.
3. Samples of species with small individuals need to be packed pooled into a food grade glass jar of 150 ml, which is not filled to the top; up to 100 ml, to avoid contact with the lock. Here, a procedural control is required (filtered water exposed to the same conditions as the samples, which can be brought to the ship frozen in such a jar). Unfiltered tap or Millipore water is not suited for this purpose. Long term storage of non-sterilized water at room temperature may lead to growth of undesired organisms and is not recommended. If handling such samples, reduce number of persons handling the sample.

- Persons close to the sample should not wear plastic clothes that easily shed plastic fibres, such as knitted or fleece clothes. Be aware that many clothes of mainly wool do contain a certain percentage plastic. Those should also be avoided.
- Plastic gloves should be either avoided or of Nitrile.
- Keep samples covered with non-plastic material whenever possible for dust protection.
- Every potential plastic contamination source (look for abrasion potential) should be sampled, sent in a glass jar with the samples, and commented in the trawl form.
- If samples are exposed to surfaces, dust wipes should be taken and sent in a glass jar with the samples.

1. Keep the samples cool at all times. Samples should be stored in the refrigerator pending measurements of weight and length and packing. Samples collected in glass jars should be stored in an upright position. All samples should be deep frozen as soon as possible.
2. Keep samples from the same area stored together in larges bags or boxes.
3. The trawl forms are to be folded twice with the writing on the inside and placed into the larger bag together with the samples. This is very important in order to assign each sample to the correct species, sampling location, sample date etc.
